# Performance of Test Supermartingale Confidence Intervals for the Success Probability of Bernoulli Trials

**DOI:** 10.6028/jres.125.003

**Published:** 2020-02-05

**Authors:** Peter Wills, Emanuel Knill, Kevin Coakley, Yanbao Zhang

**Affiliations:** 1 Department of Applied Mathematics, University of Colorado Boulder, Boulder, CO 80309 USA; 2 National Institute of Standards and Technology, Boulder, CO 80305 USA; 3 Center for Theory of Quantum Matter, University of Colorado Boulder, Boulder, CO 80309 USA; 4 Institute for Quantum Computing and Department of Physics and Astronomy, University of Waterloo, Waterloo, Ontario N2L 3G1 Canada; 5 NTT Basic Research Laboratories, NTT Corporation, 3-1 Morinosato-Wakamiya, Atsugi, Kanagawa 243-0198 Japan

**Keywords:** asymptotics, Bernoulli trials, Chernoff-Hoeffding bounds, confidence intervals, hypothesis tests, large deviations, p-values, test supermartingales

## Abstract

Given a composite null hypothesis ${ℋ}_{0}$, test supermartingales are non-negative supermartingales with respect to ${ℋ}_{0}$ with an initial value of 1. Large values of test supermartingales provide evidence against ${ℋ}_{0}$. As a result, test supermartingales are an effective tool for rejecting ${ℋ}_{0}$, particularly when the p-values obtained are very small and serve as certificates against the null hypothesis. Examples include the rejection of local realism as an explanation of Bell test experiments in the foundations of physics and the certification of entanglement in quantum information science. Test supermartingales have the advantage of being adaptable during an experiment and allowing for arbitrary stopping rules. By inversion of acceptance regions, they can also be used to determine confidence sets. We used an example to compare the performance of test supermartingales for computing p-values and confidence intervals to Chernoff-Hoeffding bounds and the “exact” p-value. The example is the problem of inferring the probability of success in a sequence of Bernoulli trials. There is a cost in using a technique that has no restriction on stopping rules, and, for a particular test supermartingale, our study quantifies this cost.

## Introduction

Experiments in physics require very high confidence to justify claims of discovery or to unambiguously exclude alternative explanations [1]. Particularly striking examples in the foundations of physics are experiments to demonstrate that theories based on local hidden variables, called local realist (LR) theories, cannot explain the statistics observed in quantum experiments called Bell tests. See Ref. [2] for a review and Refs. [3-6] for the most definitive experiments to date. Successful Bell tests imply the presence of some randomness in the observed statistics. As a result, one of the most notable applications of Bell tests is to randomness generation [7]. In this application, it is necessary to certify the randomness generated, and these certificates are equivalent to extremely small significance levels in an appropriately formulated hypothesis test. In general, such extreme significance levels are frequently required in protocols for communication or computation to ensure performance.

Bell tests consist of a sequence of “trials”, each of which gives a result $M_{i}$. LR models restrict the statistics of the $M_{i}$ and therefore constitute a composite null hypothesis to be rejected. Traditionally, data has been analyzed by estimating the value of a Bell function $\hat{B}$ and its standard error $\hat{\sigma }$ from the collective result statistics (see [8,9]). Under the null hypothesis, $\hat{B}$ is expected to be negative, so a large value of $\hat{B}$ compared to $\hat{\sigma }$ is considered to be strong evidence against the null hypothesis. This method suffers from several problems, including the failure of the Gaussian approximation in the extreme tails and the fact that the trials are observably not independent and identically distributed (i.i.d.) [9].

For the earliest discussion of martingales for analysis of Bell test experiments, see Refs. [10, 11]. In Ref. [9] a method was introduced that can give rigorous p-value bounds against LR. These p-value bounds are memory-robust, that is, without any assumptions on dependence of trial statistics on earlier trials. The method can be seen as an application of test supermartingales as defined in Ref. [12]. Test supermartingales were first considered, and many of their basic properties were proved, by Ville [13] in the same work that introduced the notion of martingales. The method involves constructing a non-negative stochastic process $V_{i}$ determined by ${\left(M_{j}\right)}_{j\leq i}$ such that the initial value is $V_{0}=1$ and, under LR models, the expectations conditional on all past events are non-increasing. As explained further below, the final value of $V_{i}$ in a sequence of n trials has expectation bounded by 1, so its inverse $p=1/V_{n}$ is a p-value bound according to Markov’s inequality. A large observed value of such a test supermartingale thus provides evidence against LR models. Refs. [9, 14] give methods to construct $V_{i}$ that achieve asymptotically optimal gain rate 𝔼⁢(-log⁡(p)/n) for i.i.d. trials, where 𝔼⁢(…) is the expectation functional. This is typically an improvement over other valid memory-robust Bell tests. Additional benefits are that $V_{i}$ can be constructed adaptively based on the observed statistics, and the p-value bounds remain valid even if the experiment is stopped based on the current value of $V_{i}$. These techniques were successfully applied to experimental data from a Bell test with photons where other methods fail [15].

Although the terminology is apparently relatively recent, test supermartingales have traditionally played a major theoretical role. Carefully constructed test supermartingales contribute to the asymptotic analysis of distributions and proofs of large deviation bounds. They can be constructed for any convex-closed null hypothesis viewed as a set of distributions, so they can be used for memory- and stopping-robust adaptive hypothesis tests in some generality. The application to Bell tests shows that at least in a regime where high significance results are required, test supermartingales can perform well or better than other methods. Here we compare the performance of test supermartingales directly to (1) the standard large deviation bounds based on the Chernoff-Hoeffding inequality [16, 17], and (2) “exact” p-value calculations. Our comparison is for a case where all calculations can be performed efficiently, namely for testing the success probability in Bernoulli trials. The three p-value bounds thus obtained have asymptotically optimal gain rates. Not surprisingly, for any given experiment, test supermartingales yield systematically worse p-value bounds, but the difference is much smaller than the experiment-to-experiment variation. This effect can be viewed as the cost of robustness against arbitrary stopping rules. For ease of calculation, we do not use an optimal test supermartingale construction, but we expect similar results no matter which test supermartingale is used.

Any hypothesis test parametrized by ϕ can be used to construct confidence regions for ϕ by acceptance region inversion (see Ref. [18], Sec. 7.1.2). Motivated by this observation, we consider the use of test supermartingales for determining confidence regions. We expect that they perform well in the high-confidence regime, with an increase in region size associated with robustness against stopping rules. We therefore compared the methods mentioned above for determining confidence intervals for the success probability in Bernoulli trials. After normalizing the difference between the interval endpoints and the success probability by the standard deviation, which is $O\,\left(1/\sqrt{n}\right)$, we find that while large deviation bounds and exact regions differ by a constant at fixed confidence levels, the test supermartingale’s normalized endpoint deviation is $\Omega \,\left(\sqrt{\log\left(n\right)}\right)$ instead of O⁢(1). This effect was noted in Ref. [12] and partially reflects a suboptimal choice of supermartingale. To maintain robustness against stopping rule, one expects $\Omega \,\left(\sqrt{\log\log\left(n\right)}\right)$ according to the law of the iterated logarithm. However, we note that if the number of trials n is fixed in advance, the normalized endpoint deviation can be reduced to O⁢(1) with an adaptive test supermartingale. So although the increase in confidence region necessitated by stopping rule robustness is not so large for reasonably sized n, when n is known ahead of time it can, in principle, be avoided without losing the ability to adapt the test supermartingale on the fly during the experiment in non-i.i.d. situations.

The remainder of the paper is structured as follows. We establish the notation to be used and define the basic concepts in Sec. 2. Here we also explain how adaptivity can help reject hypotheses for stochastic processes. We introduce the three methods to be applied to Bernoulli trials in Sec. 3. Here we also establish the basic monotonicity properties and relationships of the three p-value bounds obtained. In Sec. 4 we determine the behavior of the p-value bounds in detail, including their asymptotic behavior. In Sec. 5 we introduce the confidence intervals obtained by acceptance region inversion. We focus on one-sided intervals determined by lower bounds but note that the results apply to two-sided intervals. The observations in Secs. 4 and 5 are based on theorems whose proofs can be found in the Appendix. While many of the observations in these sections can ignore asymptotically small terms, the results in the Appendix uncompromisingly determine interval bounds for all relevant expressions, with explicit constants. Concluding remarks can be found in Sec. 6. 

## Basic Concepts

We use the usual conventions for random variables (RVs) and their values. RVs are denoted by capital letters such as X,Y,… and their values by the corresponding lower case letters x,y,…. All our RVs are finite valued. Probabilities and expectations are denoted by ℙ⁢(…) and 𝔼⁢(…), respectively. For a formula ϕ, the expression {ϕ} refers to the event where the formula is true. The notation μ⁢(X) refers to the distribution of X induced on its space of values. We use the usual conventions for conditional probabilities and expectations. Also, μ⁢(X|ϕ) denotes the probability distribution induced by X conditional on the event {ϕ}.

We consider stochastic sequences of RVs such as $𝐗={\left(X_{i}\right)}_{i=1}^{n}$ and ${𝐗}_{\leq k}={\left(X_{i}\right)}_{i=1}^{k}$. We think of the $X_{i}$ as the outcomes from a sequence of trials. For our study, we consider $𝐁={\left(B_{i}\right)}_{i=1}^{n}$, where the $B_{i}$ are {0,1}-valued RVs. The standard {0,1}-valued RV with parameter θ is the Bernoulli RV B satisfying 𝔼⁢(B)=θ. The parameter θ is also referred to as the success probability. We denote the distribution of B by $\nu_{\theta }$. We define $S_{k}=\sum_{i=1}^{k}B_{i}$ and ${\hat{\Theta }}_{k}=S_{k}/k$. We extend the RV conventions to the Greek letter ${\hat{\Theta }}_{k}$. That is, ${\hat{\theta }}_{k}=s_{k}/k=\sum_{i=1}^{k}b_{i}/k$ is the value of the RV ${\hat{\Theta }}_{k}$ determined by the values $b_{i}$ of $B_{i}$. We may omit subscripts on statistics such as $S_{n}$ and ${\hat{\Theta }}_{n}$ when they are based on the full set of n samples. Some expressions involving ${\hat{\Theta }}_{n}$ require that $n\,{\hat{\Theta }}_{n}$ is an integer, which is assured by the definition.

A null hypothesis for X is equivalent to a set ${ℋ}_{0}$ of distributions of X, which we refer to as the “null”. For our study of Bernoulli RVs, we consider the nulls

$${ℬ}_{\varphi }=\left\{\nu_{\theta }|\theta \leq \varphi \right\}$$

parametrized by 0≤φ≤1. This is the set of distributions of Bernoulli RVs with ℙ(B=1)≤φ. One can test the null hypothesis based on special statistics called p-value bounds. A statistic P=P⁢(X)≥0 is a p-value bound for ${ℋ}_{0}$ if for all $\mu \in {ℋ}_{0}$ and p≥0, ${\mathbb{P}}_{\mu }\left(P\leq p\right)\leq p$. Here, the subscript μ on ${\mathbb{P}}_{\mu }\,\left(\ldots \right)$ indicates the distribution with respect to which the probabilities are to be calculated. We usually just write “p-value” instead of “p-value bound”, even when the bounds are not achieved by a member of ${ℋ}_{0}$. Small p-values are strong evidence against the null. Since we are interested in very small p-values, we preferentially use their negative logarithm -log⁡(P) and call this the log⁡(p)-value. In this work, logarithms are base e by default.

A general method for constructing p-values is to start with an arbitrary real-valued RV Q jointly distributed with X. Usually Q is a function of X. Define the worst-case tail probability of Q as $P\left(q\right)=\sup_{\mu \in {ℋ}_{0}}{\mathbb{P}}_{\mu }\left(Q\geq q\right)$. Then P⁢(Q) is a p-value for ${ℋ}_{0}$. The argument is standard. Define $F_{\mu }\left(q\right)={\mathbb{P}}_{\mu }\left(Q\geq q\right)$. The function $F_{\mu }$ is non-increasing. We need to show that for all $\mu \in {ℋ}_{0}$, ${\mathbb{P}}_{\mu }\left(P\left(Q\right)\leq p\right)\leq p$. Since $F_{\mu }\,\left(q\right)\leq P\,\left(q\right)$, we have ${\mathbb{P}}_{\mu }\left(P\left(Q\right)\leq p\right)\leq {\mathbb{P}}_{\mu }\left(F_{\mu }\left(Q\right)\leq p\right)$. The set $\left\{q:F_{\mu }\,\left(q\right)\leq p\right\}$ is either of the form $\left[q_{\min},\infty \right)$ or $\left(q_{\min},\infty \right)$ for some $q_{\min}$. In the first case, ${\mathbb{P}}_{\mu }\left(F_{\mu }\left(Q\right)\leq p\right)={\mathbb{P}}_{\mu }\left(Q\geq q_{\min}\right)=F_{\mu }\left(q_{\min}\right)\leq p$. In the second, ${\mathbb{P}}_{\mu }\left(F_{\mu }\left(Q\right)\leq p\right)={\mathbb{P}}_{\mu }\left({⋃}_{n}\left\{q:q\geq q_{\min}+1/n\right\}\right)=\lim_{n}{\mathbb{P}}_{\mu }\left(\left\{q:q\geq q_{\min}+1/n\right\}\right)=\lim_{n}{\mathbb{P}}_{\mu }\left(F_{\mu }\left(Q\right)\leq q_{\min}+1/n\right)\leq p$, with σ-additivity applied to the countable monotone union. 

When referring to ${ℋ}_{0}$ as a null for 𝐗, we mean that ${ℋ}_{0}$ consists of the distributions where the $X_{i}$ are i.i.d., with $X_{i}$ distributed according to μ for some fixed μ independent of i. To go beyond i.i.d., we extend ${ℋ}_{0}$ to the set of distributions of 𝐗 that have the property that for all ${𝐱}_{\leq i-1}$, $\mu \left(X_{i}|{𝐗}_{\leq i-1}={𝐱}_{\leq i-1}\right)=\mu_{i}$ for some $\mu_{i}\in {ℋ}_{0}$, where $\mu_{i}$ depends on i and ${𝐱}_{\leq i-1}$. We denote the extended null by $\bar{{ℋ}_{0}}$. In particular,

$$\bar{{ℬ}_{\varphi }}=\left\{\mu :\text{for all }i\text{ and }{𝐛}_{\leq i-1}\text{, }\mu \left(B_{i}|{𝐁}_{\leq i-1}={𝐛}_{\leq i-1}\right)=\nu_{\theta }\text{ for some }\theta \leq \varphi \right\}.$$

The LR models mentioned in the introduction constitute a particular null ${ℋ}_{\mathrm{LR}}$ for sequences of trials called Bell tests. In Ref. [9], a technique called the probability-based ratio (PBR) method was introduced to construct p-values $P_{n}$ that achieve asymptotically optimal gain rates defined as $𝔼\,\left(\log\left(1/P_{n}\right)\right)/n$. The method is best understood as a way of constructing a test supermartingale for ${ℋ}_{\mathrm{LR}}$. A test supermartingale of 𝐗 for ${ℋ}_{0}$ is a stochastic sequence $𝐓={\left(T_{i}\right)}_{i=0}^{n}$ where $T_{i}$ is a function of ${𝐗}_{\leq i}$, $T_{0}=1$, $T_{i}\geq 0$ and for all $\mu \in {ℋ}_{0}$, ${𝔼}_{\mu }\,\left(T_{i+1}|{𝐗}_{\leq i}\right)\leq T_{i}$. In this work, to avoid unwanted boundary cases, we further require $T_{i}$ to be positive. The definition of test supermartingale used here is not the most general one because we consider only discrete time and avoid the customary increasing sequence of σ-algebra by making it dependent on an explicit stochastic sequence 𝐗. Every test supermartingale defines a p-value by $P_{n}=1/T_{n}$. This follows from $𝔼\,\left(T_{n}\right)\leq T_{0}=1$ (one of the characteristic properties of supermartingales) and Markov’s inequality for non-negative statistics, according to which $\mathbb{P}\left(T_{n}\geq \kappa \right)\leq 𝔼\left(T_{n}\right)/\kappa \leq 1/\kappa$. From martingale theory, the stopped process $T_{\tau }$ for any stopping rule τ with respect to 𝐗 also defines a p-value by $P=1/T_{\tau }$. Further, $P_{n}^{*}=1/\max_{i=1}^{n}T_{i}$ also defines a p-value. See Ref. [12] for a discussion and examples.

A test supermartingale 𝐓 can be viewed as the running product of the $F_{i}=T_{i}/T_{i-1}$, which we call the test factors of 𝐓. The defining properties of 𝐓 are equivalent to having $F_{i}>0$ and $𝔼\,\left(F_{i}|{𝐗}_{\leq i-1}\right)\leq 1$ for all distributions in the null, for all i. The PBR method adaptively constructs $F_{i}$ as a function of the next trial outcome $X_{i}$ from the earlier trial outcomes ${𝐗}_{\leq i-1}$. The method is designed for testing $\bar{{ℋ}_{0}}$ for a closed convex null ${ℋ}_{0}$, where asymptotically optimally gain rates are achieved when the trials are i.i.d. with a trial distribution ν not in ${ℋ}_{0}$. If ν were known, the optimal test factor would be given by x↦ν⁢(x)/μ⁢(x), where $\mu \in {ℋ}_{0}$ is the distribution in ${ℋ}_{0}$ closest to ν in Kullback-Leibler (KL) divergence $\mathrm{KL}\,\left(\nu |\mu \right)=\sum_{x}\nu \,\left(x\right)\,\log\left(\nu \,\left(x\right)/\mu \,\left(x\right)\right)$ [19]. Since ν is not known, the PBR method obtains an empirical estimate $\hat{\nu }$ of ν from ${𝐱}_{\leq i-1}$ and other information available before the i’th trial. It then determines the KL-closest $\mu \in {ℋ}_{0}$ to $\hat{\nu }$. The test factor $F_{i}$ is then given by $F_{i}\,\left(x\right)=\hat{\nu }\,\left(x\right)/\mu \,\left(x\right)$. The test factors satisfy ${𝔼}_{\mu^{\prime }}\,\left(F_{i}\right)\leq 1$ for all $\mu^{\prime }\in {ℋ}_{0}$, see Ref. [9] for a proof and applications to the problem of testing LR.

The ability to choose test factors adaptively helps reject extended nulls when the distributions vary as the experiment progresses, both when the distributions are still independent (so only the parameters vary) and when the parameters depend on past outcomes. Suppose that the distributions are sufficiently stable so that the empirical frequencies over the past k trials are statistically close to the next trial’s probability distribution. Then we can adaptively compute the test factor to be used for the next trial from the past ktrials’ empirical frequencies, for example by following the strategy outlined in the previous paragraph. The procedure now has an opportunity to reject an extended null provided only that there is a sufficiently long period where the original null does not hold. For example, consider the extended null $\bar{{ℬ}_{\varphi }}$. The true success probabilities $\theta_{i}$ at the i’th trial may vary, maybe as a result of changes in experimental parameters that need to be calibrated. Suppose that the goal is to calibrate for $\theta_{i}>\varphi$. If we use adaptive test factors and find at some point that we cannot reject $\bar{{ℬ}_{\varphi }}$ according to the running product of the test factors, we can recalibrate during the experiment. If the the recalibration succeeds at pushing $\theta_{i}$ above φ for the remaining trials, we may still reject the extended null by the end of the experiment. In many cases, the analysis is performed after the experiment, or it may not be possible to stop the experiment for recalibration. For this situation, if the frequencies for a run of k trials clearly show that $\theta_{i}<\varphi$, the adaptive test factors chosen would tend to be trivial (equal to 1), in which case the next trials do not contribute to the final test factor product. This is in contrast to a hypothesis test based on the final sum of the outcomes for which all trials contribute equally.

Let φ be a parameter of distributions of X. Here, φ need not determine the distributions. There is a close relationship between methods for determining confidence sets for φ and hypothesis tests. Let ${ℋ}_{\varphi }$ be a null such that for all distributions μ with parameter φ, $\mu \in {ℋ}_{\varphi }$. Given a family of hypothesis tests with p-values $P_{\varphi }$ for ${ℋ}_{\varphi }$, we can construct confidence sets for φ by inverting the acceptance region of $P_{\varphi }$, see Ref. [18], Sec. 7.1.2. According to this construction, the confidence set $C_{a}$ at level a is given by $\left\{\varphi |P_{\varphi }\,\left(X\right)\geq a\right\}$ and is a random quantity. The defining property of a level a confidence set is that its coverage probability satisfies ${\mathbb{P}}_{\mu }\left(\varphi \in C_{a}\right)\geq 1-a$ for all distributions $\mu \in {ℋ}_{\varphi }$. When we use this construction for sequences 𝐁 of i.i.d. Bernoulli RVs with the null ${ℬ}_{\varphi }$, we obtain one-sided confidence intervals of the form $\left[\varphi_{0},1\right]$ for $\theta =𝔼\,\left(B_{i}\right)$. When the confidence set is a one-sided interval of this type, we refer to $\varphi_{0}$ as the confidence lower bound or endpoint. If 𝐁 has a distribution μ that is not necessarily i.i.d., we can define $\Theta_{\max}=\max_{i\leq n}{𝔼}_{\mu }\,\left(B_{i}|{𝐁}_{\leq i-1}\right)$. If we use acceptance region inversion with the extended null $\bar{{ℬ}_{\varphi }}$, we obtain a confidence region for $\Theta_{\max}$. Note that $\Theta_{\max}$ is an RV whose value is covered by the confidence set with probability at least 1-a. The confidence set need not be an interval in general, but including everything between its infimum and its supremum increases the coverage probability, so the set can be converted into an interval if desired.

We focus on construction of one-sided confidence intervals. Given this machinery, we then construct two-sided intervals. For our example, we can obtain confidence upper bounds at level a by symmetry, for example by relabeling the Bernoulli outcomes 0↦1 and 1↦0. To obtain a two-sided interval at level a, we compute lower and upper bounds at level a/2. The two-sided interval is the interval between the bounds. The coverage probability of the two-sided interval is valid according to the union bound applied to maximum non-coverage probabilities of the two one-sided intervals.

## Bernoulli Hypothesis Tests

We compare three hypothesis tests for the nulls ${ℬ}_{\varphi }$ or the extended nulls $\bar{{ℬ}_{\varphi }}$: The “exact” test with p-value $P_{X}$, the Chernoff-Hoeffding test with p-value $P_{\mathrm{CH}}$ and a PBR test with p-value $P_{\mathrm{PBR}}$. In discussing properties of these tests with respect to the hypothesis parameter φ, the true success probability θ and the empirical success probability $\hat{\Theta }$, we generally assume that these parameters are in the interior of their range. In particular, 0<φ<1, 0<θ<1, and $0<\hat{\Theta }<1$. When discussing purely functional properties with respect to values $\hat{\theta }$ of $\hat{\Theta }$, we use the variable t instead of $\hat{\theta }$. By default n⁢t is a positive integer.

The p-value for the exact test is obtained from the tail for i.i.d. Bernoulli RVs:

PX,n⁢(Θ^|φ)=∑k≥Θ^⁢n(nk)⁢φk⁢(1-φ)n-k,

where $\hat{\Theta }=S_{n}/n=\sum_{i=1}^{n}B_{i}/n$ as defined in Sec. 2. Note that unlike the other p-values we consider, $P_{X,n}$ is not just a p-value bound. It is achieved by a member of the null. The quantity $P_{X,n}\,\left(t|\varphi \right)$ is decreasing as a function of t, given 0<φ<1. It is smooth and monotonically increasing as a function of φ, given t>0. To see this, compute

This is positive for φ∈(0,1). The probability that $S_{n}\geq t\,n$, given that all $B_{i}$ are distributed as $\nu_{\theta }$ with θ≤φ, is bounded by $P_{X,n}\,\left(t|\theta \right)\leq P_{X,n}\,\left(t|\varphi \right)$. That $P_{X}$ is a p-value for the case where the null is restricted to i.i.d. distributions now follows from the standard construction of p-values from worst-case (over the null) tails of statistics (here $S_{n}$) as explained in the previous section. That $P_{X}$ is a p-value for the extended null $\bar{{ℬ}_{\varphi }}$ follows from the observations that the tail probabilities of $S_{n}$ are linear functions of the distribution parameters $\theta_{1},\theta_{2},\ldots ,\theta_{n}$ where $\theta_{i}\leq \varphi ,i=1,2,\ldots ,n$, the extremal distributions in $\bar{{ℬ}_{\varphi }}$ have $B_{i}$ independent with $\mathbb{P}\left(B_{i}=1\right)=\theta_{i}\leq \varphi$, and the tail probabilities of $S_{n}$ are monotonically increasing in $\mathbb{P}\left(B_{i}=1\right)$ for each i separately. See section C of Appendix of Ref. [20].

Define ${\hat{\Theta }}_{\max}=\max\left(\hat{\Theta },\varphi \right)$. The p-value for the Chernoff-Hoeffding test is the optimal Chernoff-Hoeffding bound [16, 17] for a binary random variable given by

$$P_{\mathrm{CH},n}\,\left(\hat{\Theta }|\varphi \right)={\left(\frac{\varphi }{\Theta_{\max}}\right)}^{n\,\Theta_{\max}}\,{\left(\frac{1-\varphi }{1-\Theta_{\max}}\right)}^{n\,\left(1-\Theta_{\max}\right)}$$
$$P_{\mathrm{CH},n}\,\left(\hat{\Theta }|\varphi \right)$$
$$={\left(\frac{\varphi }{\Theta_{\max}}\right)}^{n\,\Theta_{\max}}\,{\left(\frac{1-\varphi }{1-\Theta_{\max}}\right)}^{n\,\left(1-\Theta_{\max}\right)}$$

$$=\left\{ \begin{array}{cc} {\left(\frac{\varphi }{\hat{\Theta }}\right)}^{n\,\hat{\Theta }}\,{\left(\frac{1-\varphi }{1-\hat{\Theta }}\right)}^{n\,\left(1-\hat{\Theta }\right)} & \text{if }\hat{\Theta }\geq \varphi \text{,}\\ 1 & \text{otherwise.} \end{array}\right.$$
$$=\left\{ \begin{array}{cc} {\left(\frac{\varphi }{\hat{\Theta }}\right)}^{n\,\hat{\Theta }}\,{\left(\frac{1-\varphi }{1-\hat{\Theta }}\right)}^{n\,\left(1-\hat{\Theta }\right)} & \text{if }\hat{\Theta }\geq \varphi \text{,}\\ 1 & \text{otherwise.} \end{array}\right.$$

This is a p-value for our setting because $P_{\mathrm{CH},n}\,\left(t|\varphi \right)\geq P_{X,n}\,\left(t|\varphi \right)$, see Ref. [17]. For φ≤t, we have $-\log\left(P_{\mathrm{CH},n}\,\left(t|\varphi \right)\right)=n\,\mathrm{KL}\,\left(\nu_{t}|\nu_{\varphi }\right)$. We abbreviate $\mathrm{KL}\,\left(\nu_{t}|\nu_{\varphi }\right)$ by KL⁢(t|φ). For φ≤t<1, $P_{\mathrm{CH},n}\,\left(t|\varphi \right)$ is monotonically increasing in φ, and decreasing in t. For 0≤t≤φ, it is constant.

The p-value for the PBR test that we use for comparison is constructed from a p-value for the point null $\left\{\nu_{\varphi }\right\}$ defined as

PPBR,n0⁢(Θ^|φ)=φn⁢Θ^⁢(1-φ)n⁢(1-Θ^)⁢(n+1)⁢(nn⁢Θ^).
$$P_{\mathrm{PBR},n}^{0}\,\left(\hat{\Theta }|\varphi \right)$$
=φn⁢Θ^⁢(1-φ)n⁢(1-Θ^)⁢(n+1)⁢(nn⁢Θ^).

The PBR test’s p-value for $\bar{{ℬ}_{\varphi }}$ is

$$P_{\mathrm{PBR},n}\,\left(\hat{\Theta }|\varphi \right)=\max_{0\leq \varphi^{\prime }\leq \varphi }P_{\mathrm{PBR},n}^{0}\,\left(\hat{\Theta }|\varphi^{\prime }\right).$$
$$P_{\mathrm{PBR},n}\,\left(\hat{\Theta }|\varphi \right)$$
$$=\max_{0\leq \varphi^{\prime }\leq \varphi }P_{\mathrm{PBR},n}^{0}\,\left(\hat{\Theta }|\varphi^{\prime }\right).$$

That $P_{\mathrm{PBR}}$ is a p-value for $\bar{{ℬ}_{\varphi }}$ is shown below. As a function of φ, $P_{\mathrm{PBR},n}^{0}\,\left(t|\varphi \right)$ has an isolated maximum at φ=t. This can be seen by differentiating $\log\left(\varphi^{t}\,{\left(1-\varphi \right)}^{1-t}\right)=t\,\log\left(\varphi \right)+\left(1-t\right)\,\log\left(1-\varphi \right)$. Thus in Eq. 7 when $\varphi \geq \hat{\Theta }$, the maximum is achieved by $\varphi^{\prime }=\hat{\Theta }$. We can therefore write Eq. 7 as

$$P_{\mathrm{PBR},n}\,\left(\hat{\Theta }|\varphi \right)=\left\{ \begin{array}{cc} P_{\mathrm{PBR},n}^{0}\,\left(\hat{\Theta }|\varphi \right) & \text{if }\hat{\Theta }\geq \varphi \text{,}\\ P_{\mathrm{PBR},n}^{0}\,\left(\hat{\Theta }|\hat{\Theta }\right) & \text{otherwise.} \end{array}\right.$$
$$P_{\mathrm{PBR},n}\,\left(\hat{\Theta }|\varphi \right)$$
$$=\left\{ \begin{array}{cc} P_{\mathrm{PBR},n}^{0}\,\left(\hat{\Theta }|\varphi \right) & \text{if }\hat{\Theta }\geq \varphi \text{,}\\ P_{\mathrm{PBR},n}^{0}\,\left(\hat{\Theta }|\hat{\Theta }\right) & \text{otherwise.} \end{array}\right.$$

By definition, $P_{\mathrm{PBR},n}\,\left(t|\varphi \right)$ is non-decreasing in φ and strictly increasing for φ≤t. As a function of t, it is strictly decreasing for t≥φ (integer-valued n⁢t). To see this, consider k=n⁢t≥n⁢φ and compute the ratio of successive values as follows:

$$P_{\mathrm{PBR},n}^{0}\,\left(\left(k+1\right)/n|\varphi \right)/P_{\mathrm{PBR},n}^{0}\,\left(k/n|\varphi \right)=\frac{\varphi }{1-\varphi }\,\frac{n-k}{k+1}$$
$$P_{\mathrm{PBR},n}^{0}\,\left(\left(k+1\right)/n|\varphi \right)/P_{\mathrm{PBR},n}^{0}\,\left(k/n|\varphi \right)$$
$$=\frac{\varphi }{1-\varphi }\,\frac{n-k}{k+1}$$

$$=\frac{\varphi }{1-\varphi }\,\frac{1-t}{t+1/n}$$
$$=\frac{\varphi }{1-\varphi }\,\frac{1-t}{t+1/n}$$

$$\leq \frac{\varphi }{1-\varphi }\,\frac{1-t}{t}$$
$$\leq \frac{\varphi }{1-\varphi }\,\frac{1-t}{t}$$

≤1.
≤1.

The expression for $P_{\mathrm{PBR},n}^{0}$ is the final value of a test supermartingale obtained by constructing test factors $F_{k+1}$ from $S_{k}$. Define

$${\tilde{\Theta }}_{k}=\frac{1}{k+2}\,\left(S_{k}+1\right).$$

Thus, ${\tilde{\Theta }}_{k}$ would be an empirical estimate of θ if there were two initial trials $B_{-1}$ and $B_{0}$ with values 0 and 1, respectively. The test factors are given by

$$F_{k+1}\,\left(B_{k+1}\right)={\left(\frac{{\tilde{\Theta }}_{k}}{\varphi }\right)}^{B_{k+1}}\,{\left(\frac{1-{\tilde{\Theta }}_{k}}{1-\varphi }\right)}^{1-B_{k+1}}.$$

One can verify that ${𝔼}_{\nu_{\theta }}\,\left(F_{k+1}\right)=1$ for θ=φ. More generally, set δ=θ-φ and compute

$${𝔼}_{\nu_{\theta }}\left(F_{k+1}|{\tilde{\Theta }}_{k}=t\right)=\theta \frac{t}{\varphi }+\left(1-\theta \right)\frac{1-t}{1-\varphi }$$
$${𝔼}_{\nu_{\theta }}\left(F_{k+1}|{\tilde{\Theta }}_{k}=t\right)$$
$$=\theta \,\frac{t}{\varphi }+\left(1-\theta \right)\,\frac{1-t}{1-\varphi }$$

$$=1+\delta \,\left(\frac{t}{\varphi }-\frac{1-t}{1-\varphi }\right)$$
$$=1+\delta \,\left(\frac{t}{\varphi }-\frac{1-t}{1-\varphi }\right)$$

$$=1+\delta \,\frac{t-\varphi }{\varphi \,\left(1-\varphi \right)}.$$
$$=1+\delta \,\frac{t-\varphi }{\varphi \,\left(1-\varphi \right)}.$$

As designed, $T_{n}=\prod_{k=1}^{n}F_{k}$ is a test supermartingale for the point null $\left\{\nu_{\varphi }\right\}$. Theorem 5 in section  7.2 of the Appendix, establishes that $T_{n}=1/P_{\mathrm{PBR},n}^{0}\,\left(\hat{\Theta }|\varphi \right)$. The definition of $P_{\mathrm{PBR},n}\,\left(\hat{\Theta }|\varphi \right)$ as a maximum of p-values for $\nu_{\varphi^{\prime }}$ with $\varphi^{\prime }\leq \varphi$ in Eq. 7 ensures that $P_{\mathrm{PBR},n}\,\left(\hat{\Theta }|\varphi \right)$ is a p-value for ${ℬ}_{\varphi }$.

To show that $P_{\mathrm{PBR}}$ is a p-value for $\bar{{ℬ}_{\varphi }}$, we establish that for all t (integer-valued n⁢t), $P_{\mathrm{PBR},n}\,\left(t|\varphi \right)\geq P_{\mathrm{CH},n}\,\left(t|\varphi \right)$. By direct calculation for both φ≤t and t≤φ, we have

PPBR,n⁢(t|φ)/PCH,n⁢(t|φ)=tn⁢t⁢(1-t)n⁢(1-t)⁢(n+1)⁢(nn⁢t).

The expression tk⁢(1-t)k⁢(nk) is maximized at k=n⁢t as can be seen by considering ratios for successive values of k and the calculation in Eq. 9, now applied also for k<n⁢t. Therefore,

tn⁢t⁢(1-t)n⁢(1-t)⁢(n+1)⁢(nn⁢t)=∑k=0ntn⁢t⁢(1-t)n⁢(1-t)⁢(nn⁢t)≥∑k=0ntk⁢(1-t)k⁢(nk)=1.

A better choice for test factors to construct a test supermartingale to test $\bar{{ℬ}_{\varphi }}$ would be

$$T_{k+1}^{\prime }=\left\{ \begin{array}{cc} T_{k+1} & \text{if }{\tilde{\Theta }}_{k}\geq \varphi \text{,}\\ 1 & \text{otherwise.} \end{array}\right.$$

This choice ensures that ${𝔼}_{\nu_{\theta }}\,\left(F_{k+1}|{𝐁}_{\leq k}\right)\leq 1$ for all θ≤φ but the final value of the test supermartingale obtained by multiplying these test factors is not determined by $S_{n}$, which would complicate our study.

We summarize the observations about the three tests in the following theorem.

**.**

*We have*

$$P_{X}\leq P_{\mathrm{CH}}\leq P_{\mathrm{PBR}}.$$

*The three tests satisfy the following monotonicity properties for 0<φ<1 and 0<t<1 with n⁢t integer-valued:*

-
$P_{X}\,\left(t|\varphi \right)$ *is strictly increasing in* φ *and strictly decreasing as a function of* t *.*
-
$P_{\mathrm{CH}}\,\left(t|\varphi \right)$ *is strictly increasing in* φ *for* φ≤t *, constant in* φ *for* φ≥t *, strictly decreasing in* t *for* t≥φ *and constant in* t *for* t≤φ *.*
-
$P_{\mathrm{PBR}}\,\left(t|\varphi \right)$ *is strictly increasing in* φ *for* φ≤t *, constant in* φ *for* φ≥t *and strictly decreasing in* t *for* t≥φ *.*

## Comparison of p-Values

We begin by determining the relationships between $P_{X}$, $P_{\mathrm{CH}}$ and $P_{\mathrm{PBR}}$ more precisely. Since we are interested in small p-values, it is convenient to focus on the log⁡(p)-values instead and determine their differences to $O\,\left(1/\sqrt{n}\right)$. Because of the identity $-\log\left(P_{\mathrm{CH},n}\,\left(t,\varphi \right)\right)=n\,\mathrm{KL}\,\left(t|\varphi \right)$, we reference all log⁡(p)-values to $-\log\left(P_{\mathrm{CH},n}\right)$. Here we examine the differences for t≥φ determined by the following theorem:

**.**

*For 0<φ<t<1,*

$$-\log\left(P_{\mathrm{PBR},n}\,\left(t|\varphi \right)\right)=-\log\left(P_{\mathrm{CH},n}\,\left(t|\varphi \right)\right)-\frac{1}{2}\,\log\left(n+1\right)+\frac{1}{2}\,\log\left(2\,\pi \,t\,\left(1-t\right)\right)+O\,\left(\frac{1}{n}\right),$$
$$-\log\left(P_{\mathrm{PBR},n}\,\left(t|\varphi \right)\right)$$
$$=-\log\left(P_{\mathrm{CH},n}\,\left(t|\varphi \right)\right)-\frac{1}{2}\,\log\left(n+1\right)+\frac{1}{2}\,\log\left(2\,\pi \,t\,\left(1-t\right)\right)+O\,\left(\frac{1}{n}\right),$$

$$-\log\left(P_{X,n}\,\left(t|\varphi \right)\right)=-\log\left(P_{\mathrm{CH},n}\,\left(t|\varphi \right)\right)+\frac{1}{2}\,\log\left(n\right)-\log\left(\sqrt{\frac{t}{2\,\pi \,\left(1-t\right)}}\,\frac{1-\varphi }{t-\varphi }\right)+O\,\left(\frac{1}{n}\right).$$
$$-\log\left(P_{X,n}\,\left(t|\varphi \right)\right)$$
$$=-\log\left(P_{\mathrm{CH},n}\,\left(t|\varphi \right)\right)+\frac{1}{2}\,\log\left(n\right)-\log\left(\sqrt{\frac{t}{2\,\pi \,\left(1-t\right)}}\,\frac{1-\varphi }{t-\varphi }\right)+O\,\left(\frac{1}{n}\right).$$

The theorem follows from Thms. 6, 7 and Cor. 8 proven in the Appendix, where explicit interval expressions are obtained for these log⁡(p)-value differences. The order notation assumes fixed t>φ. The bounds are not uniform, see the expressions in the Appendix for details.

The most notable observation is that there are systematic gaps of log⁡(n)/2+O⁢(1) between the log⁡(p)-values. As we already knew, there is no question that the exact test is the best of the three for this simple application. While these gaps may seem large on an absolute scale, representing factors close to $\sqrt{n}$, they are in fact much smaller than the experiment-to-experiment variation of the p-values. To determine this variation, we consider the asymptotic distributions. We can readily determine that the log⁡(p)-values are asymptotically normal with standard deviations proportional to $\sqrt{n}$, which is transferred from the variance of $\hat{\Theta }$. Compared to these standard deviations the gaps are negligible. The next theorem determines the specific way in which asymptotic normality holds. Let $N\,\left(\mu ,\sigma^{2}\right)$ denote the normal distribution with mean μ and variance $\sigma^{2}$. The notation $X_{n}\overset{𝐷}{\to }N\,\left(\mu ,\sigma^{2}\right)$ means that $X_{n}$ converges in distribution to the normal distribution with mean μ and variance $\sigma^{2}$.

**.**

*Assume 0<φ<θ<1. For $P=P_{\mathrm{CH},n}$, $P=P_{\mathrm{PBR},n}$ or $P=P_{X,n}$, the log⁡(p)-value -log⁡(P) converges in distribution according to*

$$\sqrt{n}\,\left(-\log\left(P\right)/n-\mathrm{KL}\,\left(\theta |\varphi \right)\right)\overset{𝐷}{\to }N\,\left(0,\sigma_{G}^{2}\right),$$

*with*

$$\sigma_{G}^{2}=\theta \,\left(1-\theta \right)\,{\left(\log\left(\frac{\theta }{1-\theta }\,\frac{1-\varphi }{\varphi }\right)\right)}^{2}.$$

The theorem is proven in the Appendix, see Thm. 10. For the rest of the paper, we write P or $P_{n}$ for the p-values of any one of the tests when it does not matter which one.

We display the behavior described in the above theorems for n=100 and θ=0.5 in Fig. 1. We conclude that the phenomena discussed above are already apparent for small numbers of trials. For Fig. 1, we computed the quantiles of the log⁡(p)-values numerically using the formulas provided in the previous section, substituting for t the corresponding quantile of $\hat{\Theta }$ given that ℙ(B=1)=θ. To be explicit, let $t_{r,n}\,\left(\theta \right)$ be the r-quantile of $\hat{\Theta }$ defined as the minimum value $\hat{\theta }$ of $\hat{\Theta }$ satisfying $\mathbb{P}\left(\hat{\Theta }\leq \hat{\theta }\right)\geq r$. (For simplicity we do not place the quantile in the middle of the relevant gap in the distribution.) For example, $t_{0.5,n}\,\left(\theta \right)$ is the median. Then, by the monotonicity properties of the tests, the r-quantile of $-\log\left(P_{n}\,\left(\hat{\Theta }|\varphi \right)\right)$ is given by $-\log\left(P_{n}\,\left(t_{r,n}\,\left(\theta \right)|\varphi \right)\right)$.

**Figure:**
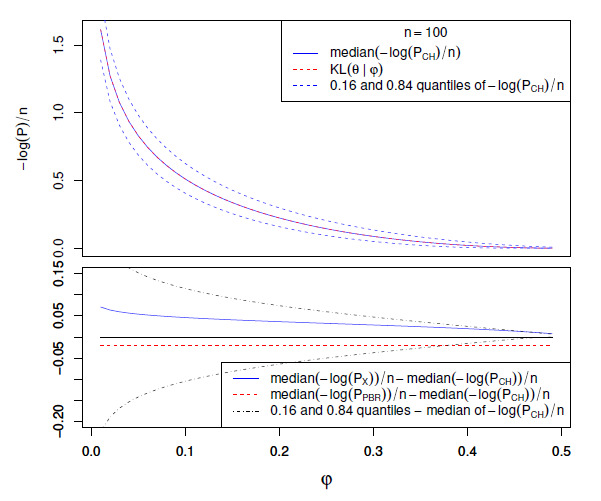
Comparison of log⁡(p)-values at n=100 and θ=0.5. The top half of the figure shows the median, and the 0.16 and 0.84 quantiles of $-\log\left(P_{\mathrm{CH},n}\,\left(\hat{\Theta }|\varphi \right)\right)/n$. For θ=0.5, the median agrees with KL⁢(θ|φ) by symmetry. The lower half shows the median differences $-\log\left(P\,\left(\hat{\Theta }|\varphi \right)\right)/n+\log\left(P_{\mathrm{CH},n}\,\left(\hat{\Theta }|\varphi \right)\right)/n$ for $P=P_{\mathrm{PBR},n}$ and $P=P_{X,n}$. The difference between the 0.16 and 0.84 quantiles and the median for $-\log\left(P_{\mathrm{CH},n}\,\left(\hat{\Theta }|\varphi \right)\right)/n$ are also shown where they are within the range of the plot; even for n as small as 100, they dominate the median differences, except where φ approaches θ=0.5, where the absolute p-values are no longer extremely small.

As noted above, the gaps between the log⁡(p)-values are of the form log⁡(n)/2+O⁢(1). In fact, it is possible to determine the asymptotic behavior of these gaps. After accounting for the explicitly given O⁢(1) terms in Thm. 2, they are asymptotically normal with variances of order O⁢(1/n). The standard deviations of the gaps are therefore small compared to their size. The precise statement of their asymptotic normality is Thm. 11 in the Appendix. Figure 2 shows how these gaps depend on the value $\hat{\theta }$ of $\hat{\Theta }$ given φ. The gaps are scaled by log⁡(n) so that they can be compared to log⁡(n)/2 visually for different values of n. The deviation from log⁡(n)/2 is most notable near the boundaries, where convergence is also slower, particularly for $P_{X}$. This behavior is consistent with the divergences as t approaches φ in the explicit interval bounds in Thm. 7 and Cor. 8.

**Figure:**
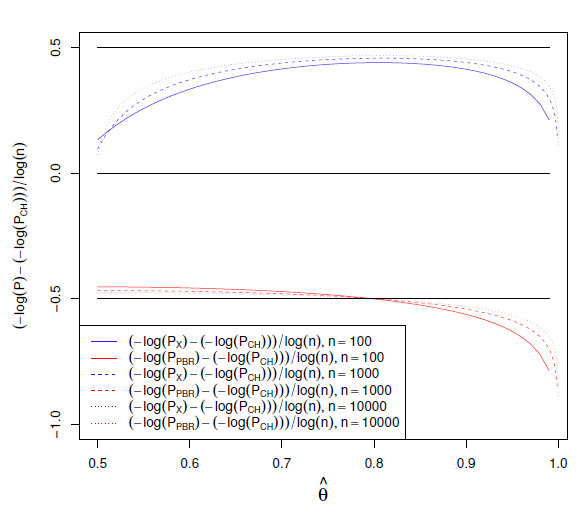
Gaps between the log⁡(p)-values depending on $\hat{\theta }$ at φ=0.5. We show the normalized differences $\left(-\log\left(P_{n}\,\left(\hat{\theta }|\varphi \right)\right)+\log\left(P_{\mathrm{CH},n}\,\left(\hat{\theta }|\varphi \right)\right)\right)/\log\left(n\right)$ for $P=P_{\mathrm{CH}}$ and $P=P_{X}$ at n=100,1000, and 10000. For large n, at constant $\hat{\theta }$ with $0.5<\hat{\theta }<1$, the PBR test’s normalized difference converges to -0.5, and the exact test’s normalized difference converges to 0.5. The horizontal lines at ±0.5 indicate this limit. The lowest order normalized asymptotic differences from ±0.5 are O⁢(1/log⁡(n)) and diverge at $\hat{\theta }=0.5$ and $\hat{\theta }=1$.

## Comparison of Confidence Intervals

Before presentation of our technical results, we remark that there are many excellent publications on construction of one-sided and two-sided confidence intervals for the success probability of binomial trials for the case of i.i.d. observations including [21] and [22].

Let P be one of $P_{\mathrm{CH},n}$, $P_{\mathrm{PBR},n}$ or $P_{X,n}$. Given a value $\hat{\theta }$ of $\hat{\Theta }$, the level-a confidence set determined by the test with p-value P is $I=\left\{\varphi |P\,\left(\hat{\theta }|\varphi \right)\geq a\right\}$. By the monotonicity properties of P, the closure of I is an interval $\left[\varphi_{a}\,\left(\hat{\theta };P\right),1\right]$. We can compute the endpoint $\varphi_{a}$ by numerically inverting the exact expressions for P. An example is shown in Fig. 3, where we show the endpoints according to each test for a=0.01 and $\hat{\theta }=0.5$ as a function of n. All tests’ endpoints converge to 0.5 as the number of trials grows. Notably, the relative separation between the endpoints is not large at level a=0.01.

**Figure:**
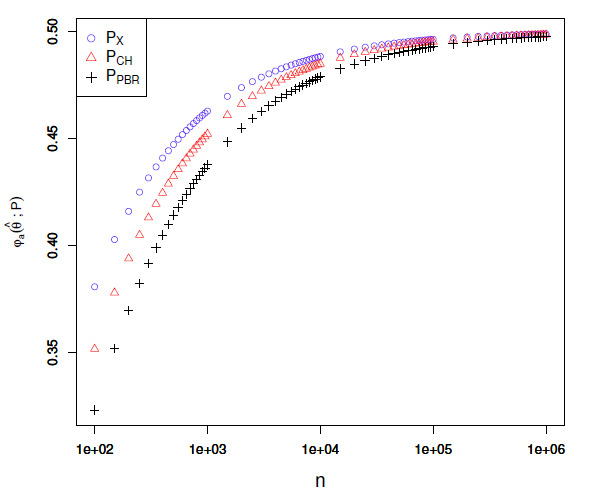
Lower endpoints for the level 0.01 confidence set of the three tests as a function of n, where $\hat{\theta }=0.5$.

To quantify the behavior of the endpoints for the different tests, we normalize by the empirical standard deviation $\hat{\sigma }=\sqrt{\hat{\theta }\,\left(1-\hat{\theta }\right)/n}$. The empirical endpoint deviation is then defined as

$$\gamma_{a}\,\left(\hat{\theta };P\right)=\frac{\hat{\theta }-\varphi_{a}\,\left(\hat{\theta };P\right)}{\hat{\sigma }}.$$

For the exact test and for large n, we expect this quantity to be determined by the tail probabilities of a standard normal distribution. That is, if the significance a is the probability that a normal RV of variance 1 exceeds κ, we expect $\gamma_{a}\,\left(\hat{\theta };P_{X}\right)\approx \kappa$.

We take the point of view that the performance of a test is characterized by the size of the endpoint deviation. If the relative size of the deviations for two tests is close to 1 then they perform similarly for the purpose of characterizing the parameter θ. Another way of comparing the intervals obtained is to consider their coverage probabilities. For our situation, the coverage probability for test P at a can be approximated by determining $a^{\prime }$ such that $\gamma_{a^{\prime }}\,\left(\theta ;P_{X}\right)=\gamma_{a}\,\left(\theta ;P\right)$. From Thm. 4 below, one can infer that the coverage probability is then approximately $1-a^{\prime }\geq 1-a$. The coverage probabilities can be very conservative (larger than 1-a), particularly for small a and $P=P_{\mathrm{PBR}}$.

We determined interval bounds for the empirical endpoint deviation for all three tests. The details are in section 7.5 of the Appendix. The next theorem summarizes the results asymptotically.

**.**

*Let $q\left(x\right)=-\log\left(P_{N\,\left(0,1\right)}\left(N\geq x\right)\right)$ be the negative logarithm of the tail of the standard normal. Fix $\hat{\theta }\in \left(0,1\right)$. Write α=|log⁡(a)|. There is a constant c (depending on $\hat{\theta }$) such that for α∈(1,c⁢n], $\gamma_{a}$ satisfies*

$$\gamma_{a}\,\left(\hat{\theta };P_{\mathrm{CH}}\right)=\sqrt{2\,\alpha }+O\,\left(\alpha /\sqrt{n}\right),$$
$$\gamma_{a}\,\left(\hat{\theta };P_{\mathrm{CH}}\right)$$
$$=\sqrt{2\,\alpha }+O\,\left(\alpha /\sqrt{n}\right),$$

$$\gamma_{a}\,\left(\hat{\theta };P_{\mathrm{PBR}}\right)=\sqrt{2\,\alpha +\log\left(n\right)/2-\log\left(2\,\pi \,\hat{\theta }\,\left(1-\hat{\theta }\right)\right)/2}+O\,\left(\alpha /\sqrt{n}\right),$$
$$\gamma_{a}\,\left(\hat{\theta };P_{\mathrm{PBR}}\right)$$
$$=\sqrt{2\,\alpha +\log\left(n\right)/2-\log\left(2\,\pi \,\hat{\theta }\,\left(1-\hat{\theta }\right)\right)/2}+O\,\left(\alpha /\sqrt{n}\right),$$

$$\gamma_{a}\,\left(\hat{\theta };P_{X}\right)=q^{-1}\,\left(\alpha \right)+O\,\left(\alpha /\sqrt{n}\right).$$
$$\gamma_{a}\,\left(\hat{\theta };P_{X}\right)$$
$$=q^{-1}\,\left(\alpha \right)+O\,\left(\alpha /\sqrt{n}\right).$$

*The last expression has the following approximation relevant for sufficiently large α:*

$$\gamma_{a}\,\left(\hat{\theta };P_{X}\right)=\sqrt{2\,\alpha -\log\left(2\,\pi \right)-\log\left(2\,\alpha -\log\left(2\,\pi \right)\right)}+O\,\left(\log\left(\alpha \right)/\alpha^{3/2}\right)+O\,\left(\alpha /\sqrt{n}\right).$$

For $\alpha =o\,\left(\sqrt{n}\right)$, the relative error of the approximation in the first two identities goes to zero as n grows. This is not the case for the last identity, where the relative error for large n is dominated by the term $O\,\left(\log\left(\alpha \right)/\alpha^{3/2}\right)$, and large α is required for a small relative error.

**Proof.**

The expression for $\gamma_{a}\,\left(\hat{\theta };P_{\mathrm{CH}}\right)$ is obtained from Thm. 12 in the Appendix by changing the relative approximation errors into absolute errors.

To obtain the expression for $\gamma_{a}\,\left(\hat{\theta };P_{\mathrm{PBR}}\right)$, note that the term Δ in Thm. 13 satisfies $\Delta =\log\left(n\right)/2-\log\left(2\,\pi \,\hat{\theta }\,\left(1-\hat{\theta }\right)\right)/2+O\,\left(1/n\right)$, see Thm. 6. The O⁢(1/n) under the square root pulls out to an $O\,\left(1/\left(\sqrt{\max\left(\alpha ,\log\left(n\right)\right)}\,n\right)\right)$ term that is dominated by $O\,\left(\alpha /\sqrt{n}\right)$ because α≥1 by assumption.

To obtain the expressions for $\gamma_{a}\,\left(\hat{\theta };P_{X}\right)$, we refer to Thm. 14, where the lower bound on α implies α≥1>log⁡(2). The intervals in Thm. 14 give relative errors that need to be converted to absolute quantities. By positivity and monotonicity of $q^{-1}$, for sufficiently large n and for some positive constants u and v, we have

$$\gamma_{a}\,\left(\hat{\theta };P_{X}\right)\in \left[q^{-1}\,\left(\alpha \,\left(1-u\,\sqrt{\alpha }/\sqrt{n}\right)\right)\,\left(1-v\,\sqrt{\alpha }/\sqrt{n}\right),q^{-1}\,\left(\alpha \,\left(1+u\,\sqrt{\alpha }/\sqrt{n}\right)\right)\,\left(1+v\,\sqrt{\alpha }/\sqrt{n}\right)\right].$$

Explicit values for u and v can be obtained from Thm. 14. We simplified the argument of $q^{-1}$ by absorbing the additive terms in the theorem into the term $u\,\alpha \,\sqrt{\alpha }/\sqrt{n}$ with the constant u chosen to be sufficiently large. Consider Eq. 94 with $\delta =u\,\sqrt{\alpha }/\sqrt{n}$. For sufficiently large n, the expression in the denominator of the approximation error on the right-hand side exceeds a constant multiple of α. From this, with some new constant $u^{\prime }$,

$$\gamma_{a}\,\left(\hat{\theta };P_{X}\right)\in \left[q^{-1}\,\left(\alpha \right)\,\left(1-u^{\prime }\,\sqrt{\alpha }/\sqrt{n}\right)\,\left(1-v\,\sqrt{\alpha }/\sqrt{n}\right),q^{-1}\,\left(\alpha \right)\,\left(1+u^{\prime }\,\sqrt{\alpha }/\sqrt{n}\right)\,\left(1+v\,\sqrt{\alpha }/\sqrt{n}\right)\right],$$

which, with order notation simplifies further to

$$\gamma_{a}\,\left(\hat{\theta };P_{X}\right)=q^{-1}\,\left(\alpha \right)\,\left(1+O\,\left(\sqrt{\alpha }/\sqrt{n}\right)\right).$$

It now suffices to apply $q^{-1}\,\left(\alpha \right)=O\,\left(\sqrt{\alpha }\right)$ (see the proof of Eq. 24 below) and Eq. 23 is obtained.

For Eq. 24, we bound $x=q^{-1}\,\left(\alpha \right)$, which we can do via bounds for α=q⁢(x). From the expression $q\,\left(x\right)=x^{2}/2+\log\left(2\,\pi \right)/2-\log\left(Y\,\left(x\right)\right)=x^{2}/2+\log\left(2\,\pi \right)/2+\log\left(x\right)-\log\left(x\,Y\,\left(x\right)\right)$ in the statement of Thm. 14 and the bounds in Eq. 58, we have the two inequalities

$$q\,\left(x\right)=x^{2}/2+\log\left(2\,\pi \right)/2+\log\left(x\right)-\log\left(x\,Y\,\left(x\right)\right)\geq x^{2}/2+\log\left(2\,\pi \right)/2+\log\left(x\right),$$
q⁢(x)
$$=x^{2}/2+\log\left(2\,\pi \right)/2+\log\left(x\right)-\log\left(x\,Y\,\left(x\right)\right)\geq x^{2}/2+\log\left(2\,\pi \right)/2+\log\left(x\right),$$

$$q\,\left(x\right)\leq x^{2}/2+\log\left(2\,\pi \right)/2+\log\left(x\right)+1/x^{2}.$$
q⁢(x)
$$\leq x^{2}/2+\log\left(2\,\pi \right)/2+\log\left(x\right)+1/x^{2}.$$

Let $l\,\left(x\right)=x^{2}/2+\log\left(2\,\pi \right)/2+\log\left(x\right)$, which is monotonically increasing, as is q. The first inequality implies that $q^{-1}\leq l^{-1}$. We need a bound of the form $q\,\left(x\right)\leq d\,x^{2}$, from which we can conclude that $x^{2}\geq \alpha /d$. A bound of this type can be obtained from Eq. 91 in the Appendix. For definiteness, we restrict to α≥6 and show that the bound holds with d=1. By Eq. 29, it suffices to establish that for $x\geq \sqrt{6}$, $l\,\left(x\right)+1/x^{2}\leq x^{2}$. Since log⁡(2⁢π)/2≤1, we have $\log\left(2\,\pi \right)/2+\log\left(x\right)+1/x^{2}\leq 1+\log\left(1+\left(x-1\right)\right)+1/x^{2}\leq x+1/x^{2}$. For x≥9/4, $x+1/x^{2}\leq x^{2}/2$. To finish the argument, apply the inequality $\sqrt{6}\geq 9/4$.

Given the bound $x^{2}\geq \alpha$, Eq. 29 becomes q⁢(x)=α≤l⁢(x)+1/α. With Eq. 28 we get α=q⁢(x)∈l⁢(x)+[0,1]/α. Equivalently,

$$l\,\left(x\right)\in \alpha +\frac{1}{\alpha }\,\left[-1,0\right].$$

Applying the monotone $l^{-1}$ on both sides gives

$$x=l^{-1}\,\left(l\,\left(x\right)\right)\in l^{-1}\,\left(\alpha +\frac{1}{\alpha }\,\left[-1,0\right]\right).$$

Let $\alpha^{\prime }$ satisfy $x=l^{-1}\,\left(\alpha^{\prime }\right)$ with $\alpha^{\prime }=\alpha +\delta$ and δ∈[-1,0]/α. Write $z=x^{2}$ and c=log⁡(2⁢π). We have $l\,\left(z^{1/2}\right)=z/2+c/2+\log\left(z\right)/2=\alpha^{\prime }$, which we can write as a fixed point equation z=f⁢(z) for z with $f\,\left(z\right)=2\,\alpha^{\prime }-c-\log\left(z\right)$. We can accomplish our goal by determining lower and upper bounds on the fixed point. Since $\frac{d}{d\,y}\,f\,\left(y\right)=-1/y<0$ for y>0, the iteration $z_{0}=2\,\alpha^{\prime }-c$ and $z_{k}=f\,\left(z_{k-1}\right)$ is alternating around the fixed point z, provided $z_{k}>0$ for all k. Provided $z_{0}>1$, $z_{1}=f\,\left(z_{0}\right)<z_{0}$, from which we conclude that $z_{1}\leq z<z_{0}$. Since we are assuming that α≥6 and from above $z_{0}\geq 2\,\left(\alpha -1/\alpha \right)-c$, the condition $z_{0}>1$ is satisfied. If $z_{1}\geq 1$, then $0>\frac{d}{d\,y}\,f\,\left(y\right)>-1$ between $z_{1}$ and $z_{0}$, which implies that $z_{0}$ and $z_{1}$ are in the region where the iteration converges to z. For our bounds, we only require $z_{1}>0$, so that we can bound z according to $z_{1}\leq z\leq z_{2}$. That $z_{1}>0$ follows from log⁡(y)<y for y>0. We have

$$z_{2}-z_{1}=z_{0}-\log\left(z_{1}\right)-\left(z_{0}-\log\left(z_{0}\right)\right)$$
$$z_{2}-z_{1}$$
$$=z_{0}-\log\left(z_{1}\right)-\left(z_{0}-\log\left(z_{0}\right)\right)$$

$$=\log\left(z_{0}/z_{1}\right)$$
$$=\log\left(z_{0}/z_{1}\right)$$

$$=\log\left(z_{0}/\left(z_{0}-\log\left(z_{0}\right)\right)\right)$$
$$=\log\left(z_{0}/\left(z_{0}-\log\left(z_{0}\right)\right)\right)$$

$$=-\log\left(1-\log\left(z_{0}\right)/z_{0}\right)$$
$$=-\log\left(1-\log\left(z_{0}\right)/z_{0}\right)$$

$$=O\,\left(\log\left(z_{0}\right)/z_{0}\right)=O\,\left(\log\left(\alpha^{\prime }\right)/\alpha^{\prime }\right)=O\,\left(\log\left(\alpha \right)/\alpha \right),$$
$$=O\,\left(\log\left(z_{0}\right)/z_{0}\right)=O\,\left(\log\left(\alpha^{\prime }\right)/\alpha^{\prime }\right)=O\,\left(\log\left(\alpha \right)/\alpha \right),$$

where $z_{0}=2\,\alpha^{\prime }-c\in 2\,\alpha -c+2\,\left[-1,0\right]/\alpha$, and so $-\log\left(z_{0}\right)=-\log\left(2\,\alpha -c\right)+O\,\left(1/\alpha^{2}\right)$. For $z_{1}$ we get $z_{1}=z_{0}-\log\left(z_{0}\right)=2\,\alpha -c-\log\left(2\,\alpha -c\right)+O\,\left(1/\alpha \right)$. Applying Eq.32 and from the definitions

$$q^{-1}\,\left(\alpha \right)=x=\sqrt{2\,\alpha -c-\log\left(2\,\alpha -c\right)+O\,\left(\log\left(\alpha \right)/\alpha \right)}.$$

The approximation error in Eq. 24 is obtained by expanding the square root. We could have used Newton’s method starting from $z_{0}$ to obtain better approximations in one step, but the resulting expression is more involved. ∎

The expression for $\gamma_{a}\,\left(\hat{\theta };P_{X}\right)$ confirms our expectation that it approaches the expected value for a standard normal distribution and may be compared to the Berry-Esseen theorem [23]. The empirical endpoint deviation of the CH test approaches that of the exact test for small a (large α). Their squares differ by a term of order log⁡(α)=log⁡|log⁡(a)|. Notably, the ratio of the PBR and CH tests’ empirical endpoint deviation grows as $\Theta \,\left(\sqrt{\log\left(n\right)/\alpha }\right)$. The relationships are visualized in Figs. 4, 5 and 6 for different values of a. The figures show that the relative sizes of the empirical endpoint deviations tend toward 1 with smaller a. The $\Theta \,\left(\sqrt{\log\left(n\right)/\alpha }\right)$ relative growth of the PBR test’s endpoint deviations leads to less than a doubling of the deviations relative to the exact test’s at a=0.01 and a=0.001 even for $n=10^{6}$. So while the test’s coverage probabilities are much closer to 1 than the nominal value of 1-a, we believe that it does not lead to unreasonably conservative results in many applications.

**Figure:**
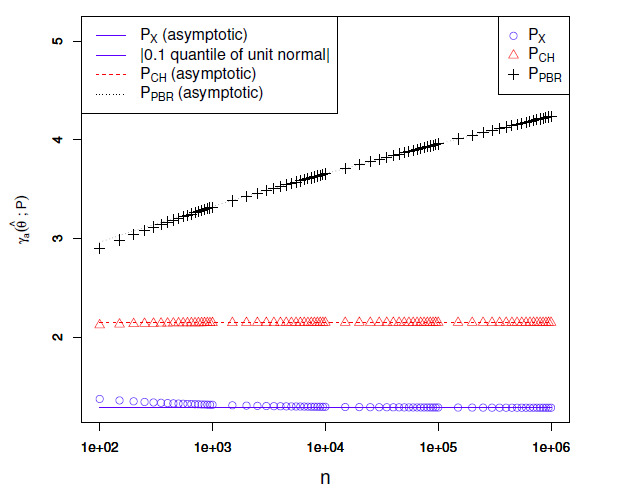
Empirical confidence set endpoint deviations at level a=0.1 for $\hat{\theta }=0.5$ as a function of n. The continuous lines show the expressions obtained after dropping the $O\,\left(1/\sqrt{n}\right)$ terms. For the exact test, these expressions are the same as the normal approximation and therefore match the absolute value of the 0.1 quantile of a unit normal.

**Figure:**
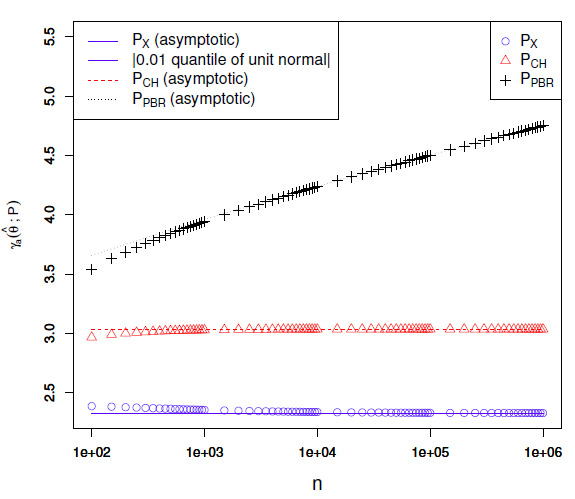
Empirical confidence set endpoint deviations at level a=0.01 for $\hat{\theta }=0.5$ as a function of n. See the caption of Fig. 4.

**Figure:**
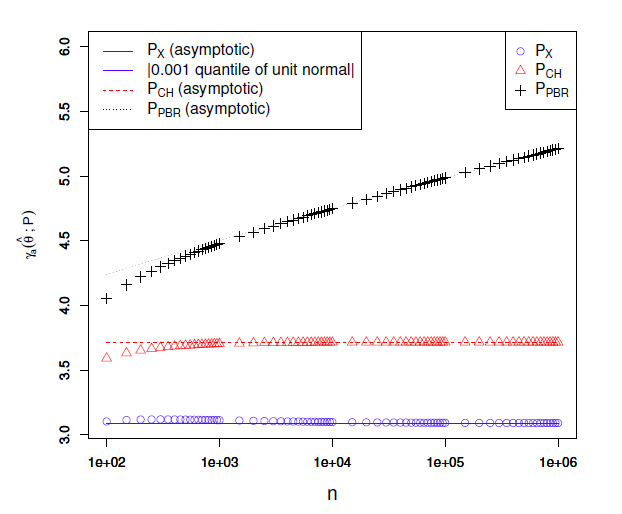
Empirical confidence set endpoint deviations at level a=0.001 for $\hat{\theta }=0.5$ as a function of n. See the caption of Fig. 4.

Next we consider the behavior of the true endpoint deviations given by the normalized difference of the true success probability θ and the endpoint obtained from one of the tests. Let $\sigma =\sqrt{\theta \,\left(1-\theta \right)/n}$ be the true standard deviation and define the true endpoint deviation determined by test P as

$${\tilde{\gamma }}_{a}\,\left(\hat{\Theta }|P\right)=\left(\theta -\varphi_{a}\,\left(\hat{\Theta }|P\right)\right)/\sigma$$
$${\tilde{\gamma }}_{a}\,\left(\hat{\Theta }|P\right)$$
$$=\left(\theta -\varphi_{a}\,\left(\hat{\Theta }|P\right)\right)/\sigma$$

$$=\left(\theta -\hat{\Theta }\right)/\sigma +\gamma_{a}\,\left(\hat{\Theta }|P\right)\,\hat{\sigma }/\sigma .$$
$$=\left(\theta -\hat{\Theta }\right)/\sigma +\gamma_{a}\,\left(\hat{\Theta }|P\right)\,\hat{\sigma }/\sigma .$$

The true endpoint deviations show how the inferred endpoint compares to θ and therefore directly exhibits the statistical fluctuations of $\hat{\Theta }$. In contrast, the empirical endpoint deviations are to lowest order independent of $\hat{\theta }-\theta$.

We take the view that two tests’ endpoints perform similarly if their true endpoint deviations differ by an amount that is small compared to the width of the distribution of the true endpoint deviations. To compare the three tests on this basis, we consider the quantiles for $\hat{\Theta }$ corresponding to ±κ Gaussian standard deviations from θ with κ constant. The quantiles satisfy $\theta_{\pm \kappa }=\theta \pm \kappa \,\sigma \,\left(1+O\,\left(1/\sqrt{n}\right)\right)$, by the Berry-Esseen theorem or from Thm. 14. Since $\hat{\sigma }=\sigma \,\left(1+O\,\left(1/\sqrt{n}\right)\right)$, we can also see that $\gamma_{a}\,\left(\theta_{\pm \kappa }|P\right)=\gamma_{a}\,\left(\hat{\theta }|P\right)+O\,\left(1/\sqrt{n}\right)$, and so by substituting into the definition,

$${\tilde{\gamma }}_{a}\,\left(\theta_{\pm \kappa }|P\right)=\gamma_{a}\,\left(\theta |P\right)\pm \kappa +O\,\left(1/\sqrt{n}\right),$$
$${\tilde{\gamma }}_{a}\,\left(\theta_{\pm \kappa }|P\right)=\gamma_{a}\,\left(\theta |P\right)\pm \kappa +O\,\left(1/\sqrt{n}\right),$$

where the implicit constants depend on κ. For large α, the CH and exact tests’ endpoints are close and are dominated by κ, so they perform similarly. But this does not hold for the comparison of the CH or the exact test’s endpoints to those of the PBR test, since the latter’s endpoint deviation grows as $\sqrt{\log\left(n\right)/2}$.

The PBR test’s robustness to stopping rules requires that endpoint deviations must grow. Qualitatively, we expect a growth of at least $\Omega \,\left(\sqrt{\log\log\left(n\right)}\right)$ due to the law of the iterated logarithm. This growth is slower than the $\sqrt{\log\left(n\right)/2}$ growth found above, suggesting that improvements are possible, as observed in Ref. [12]. In many applications, the number of trials to be acquired can be determined ahead of time, so full robustness to stopping rules is not necessary. However, the ability to adapt to changing experimental conditions may still be helpful, as the example in Sec. 2 shows. If we know the number of trials ahead of time, we can retain the ability to adapt while avoiding the asymptotic growth of the endpoint deviations of the PBR test.

A strategy for avoiding the asymptotic growth of the PBR test’s endpoint deviations is to set aside the first m=λ⁢n of the trials for training to infer the probability of success, and then use this to determine the test factor to be used on the remaining (1-λ)⁢n of the trials. With this strategy, the endpoint deviations are bounded on average and typically. We formalize the training strategy as follows: Modify Eq. 11 by setting $F_{k=1}=1$ for k<m and for k≥m,

$$F_{k+1}\,\left(B_{k+1}\right)=F\,\left(B_{k+1}\right)={\left(\frac{{\hat{\Theta }}_{m}}{\varphi }\right)}^{B_{k+1}}\,{\left(\frac{1-{\hat{\Theta }}_{m}}{1-\varphi }\right)}^{1-B_{k+1}}.$$

Let G=F if $\varphi \leq {\hat{\Theta }}_{m}$ and G=1 otherwise. The $G_{k+1}$ are valid test factors for the null ${ℬ}_{\varphi }$. A p-value for testing ${\bar{ℬ}}_{\varphi }$ is given by

$$P_{\lambda }\,\left(𝐁|\varphi \right)=G\,{\left(1\right)}^{-\left(n-m\right)\,{\hat{\Theta }}_{m}^{\prime }}\,G\,{\left(0\right)}^{-\left(n-m\right)\,\left(1-{\hat{\Theta }}_{m}^{\prime }\right)}$$
$$P_{\lambda }\,\left(𝐁|\varphi \right)$$
$$=G\,{\left(1\right)}^{-\left(n-m\right)\,{\hat{\Theta }}_{m}^{\prime }}\,G\,{\left(0\right)}^{-\left(n-m\right)\,\left(1-{\hat{\Theta }}_{m}^{\prime }\right)}$$

where ${\hat{\Theta }}_{m}^{\prime }$ is defined by $\left(n-m\right)\,{\hat{\Theta }}_{m}^{\prime }+m\,{\hat{\Theta }}_{m}=n\,{\hat{\Theta }}_{n}$. We call this the $P_{\lambda }$ test.

Define

$$Q_{\lambda }\,\left(𝐁|\varphi \right)={\left(\frac{\varphi }{{\hat{\Theta }}_{m}}\right)}^{\left(n-m\right)\,{\hat{\Theta }}_{m}^{\prime }}\,{\left(\frac{1-\varphi }{1-{\hat{\Theta }}_{m}}\right)}^{\left(n-m\right)\,\left(1-{\hat{\Theta }}_{m}^{\prime }\right)}.$$
$$Q_{\lambda }\,\left(𝐁|\varphi \right)$$
$$={\left(\frac{\varphi }{{\hat{\Theta }}_{m}}\right)}^{\left(n-m\right)\,{\hat{\Theta }}_{m}^{\prime }}\,{\left(\frac{1-\varphi }{1-{\hat{\Theta }}_{m}}\right)}^{\left(n-m\right)\,\left(1-{\hat{\Theta }}_{m}^{\prime }\right)}.$$

Then for $\varphi \leq {\hat{\Theta }}_{m}$, $Q_{\lambda }\,\left(𝐁|\varphi \right)=P_{\lambda }\,\left(𝐁|\varphi \right)$. To investigate the behavior of these quantities, we consider values 𝐛, $\hat{\theta }$, ${\hat{\theta }}_{m}$ and ${\hat{\theta }}_{m}^{\prime }$ of the corresponding RVs. As a function of φ, $Q_{\lambda }\,\left(𝐛|\varphi \right)$ is maximized at $\varphi ={\hat{\theta }}_{m}^{\prime }$ and monotone on either side of ${\hat{\theta }}_{m}^{\prime }$. If ${\hat{\theta }}_{m}\leq \varphi \leq {\hat{\theta }}_{m}^{\prime }$, then $Q_{\lambda }\,\left(𝐛|\varphi \right)\geq 1=P_{\lambda }\,\left(𝐛|\varphi \right)$, So for $\varphi \leq \max\left({\hat{\theta }}_{m},{\hat{\theta }}_{m}^{\prime }\right)$, we can use $Q_{\lambda }$ instead of $P_{\lambda }$ without changing endpoint calculations.

For determining the endpoint of a level-a one-sided confidence interval from $P_{\lambda }$, we seek the maximum φ such that for all $\varphi^{\prime }\leq \varphi$, $P_{\lambda }\,\left(𝐛|\varphi^{\prime }\right)\leq a$. This maximum value of φ satisfies that $\varphi \leq \min\left({\hat{\theta }}_{m}^{\prime },{\hat{\theta }}_{m}\right)$: For ${\hat{\theta }}_{m}\leq {\hat{\theta }}_{m}^{\prime }$, this follows from $P_{\lambda }\,\left(𝐛|{\hat{\theta }}_{m}\right)=1$. For ${\hat{\theta }}_{m}\geq {\hat{\theta }}_{m}^{\prime }$, the location of the maximum of $Q_{\lambda }$ implies that $P_{\lambda }\,\left(𝐛|{\hat{\theta }}_{m}^{\prime }\right)\geq P_{\lambda }\,\left(𝐛|{\hat{\theta }}_{m}\right)=1$.

We show that endpoint deviations from the $P_{\lambda }$ test are typically a constant factor larger than those of the CH test. For large α, the factor approaches $1/\sqrt{1-\lambda }$, approximating the endpoint deviations for a CH test with (1-λ)⁢n trials. We begin by comparing $P_{\lambda }$ to $P_{\mathrm{CH},\left(1-\lambda \right)\,n}$ with the latter applied to the last (1-λ)⁢n trials and $\varphi \leq {\hat{\theta }}_{m}^{\prime }$, where we can use $Q_{\lambda }$ in place of $P_{\lambda }$.

$$Q_{\lambda }\,\left(𝐛|\varphi \right)/P_{\mathrm{CH},\left(1-\lambda \right)\,n}\,\left({\hat{\theta }}_{m}^{\prime }|\varphi \right)={\left(\frac{{\hat{\theta }}_{m}^{\prime }}{{\hat{\theta }}_{m}}\right)}^{\left(1-\lambda \right)\,n\,{\hat{\theta }}_{m}^{\prime }}\,{\left(\frac{1-{\hat{\theta }}_{m}^{\prime }}{1-{\hat{\theta }}_{m}}\right)}^{\left(1-\lambda \right)\,n\,\left(1-{\hat{\theta }}_{m}^{\prime }\right)}.$$

Or, for the log⁡(p)-value difference $l_{p}$,

$$l_{p}=-\log\left(Q_{\lambda }\,\left(𝐛|\varphi \right)\right)+\log\left(P_{\mathrm{CH},\left(1-\lambda \right)\,n}\,\left({\hat{\theta }}_{m}^{\prime }|\varphi \right)\right)=-\left(1-\lambda \right)\,n\,\mathrm{KL}\,\left({\hat{\theta }}_{m}^{\prime }|{\hat{\theta }}_{m}\right),$$

which is non-positive. By expanding to second order,

KL⁢(t+x|t+y)=(t+x)⁢(log⁡(1+x/t)-log⁡(1+y/t))
KL⁢(t+x|t+y)
=(t+x)⁢(log⁡(1+x/t)-log⁡(1+y/t))

+(1-t-x)⁢(log⁡(1-x/(1-t))-log⁡(1-y/(1-t)))
+(1-t-x)⁢(log⁡(1-x/(1-t))-log⁡(1-y/(1-t)))

$$=\frac{{\left(x-y\right)}^{2}}{2\,t\,\left(1-t\right)}+O\left(\max{\left(|x|,|y|\right)}^{3}\right).$$
$$=\frac{{\left(x-y\right)}^{2}}{2\,t\,\left(1-t\right)}+O\left(\max{\left(|x|,|y|\right)}^{3}\right).$$

Let $\Delta ={\hat{\Theta }}_{m}-\theta$ and $\Delta^{\prime }={\hat{\Theta }}_{m}^{\prime }-\theta$. From the above expansion with t=θ, $x=\delta^{\prime }$ and y=δ (where δ and $\delta^{\prime }$ are values of Δ and $\Delta^{\prime }$)

$$l_{p}=-\left(1-\lambda \right)\,n\,\left(\frac{{\left(\delta -\delta^{\prime }\right)}^{2}}{2\,\theta \,\left(1-\theta \right)}+O\,\left(\max\left(\left|\delta \right|,{\left|\delta^{\prime }\right|}^{3}\right)\right)\right).$$
$$l_{p}$$
$$=-\left(1-\lambda \right)\,n\,\left(\frac{{\left(\delta -\delta^{\prime }\right)}^{2}}{2\,\theta \,\left(1-\theta \right)}+O\,\left(\max\left(\left|\delta \right|,{\left|\delta^{\prime }\right|}^{3}\right)\right)\right).$$

The RVs Δ and $\Delta^{\prime }$ are independent with means 0 and variances $\sigma^{2}/\lambda$ and $\sigma^{2}/\left(1-\lambda \right)$. Furthermore, $\sqrt{n}\,\Delta$ and $\sqrt{n}\,\Delta^{\prime }$ are asymptotically normal with variances θ⁢(1-θ)/λ and θ⁢(1-θ)/(1-λ). Consequently, the RV $\sqrt{n}\,\left(\Delta -\Delta^{\prime }\right)$ is asymptotically normal with variance v=θ⁢(1-θ)/(λ⁢(1-λ)). Accordingly, the probability that $n\,{\left(\Delta -\Delta^{\prime }\right)}^{2}\geq \kappa^{2}\,\theta \,\left(1-\theta \right)/\left(\lambda \,\left(1-\lambda \right)\right)$ is asymptotically given by the two-sided tail for κ standard deviations of the standard normal. For determining typical behavior, we consider ${\left(\delta -\delta^{\prime }\right)}^{2}=\kappa^{2}\,\theta \,\left(1-\theta \right)/\left(n\,\lambda \,\left(1-\lambda \right)\right)$ with κ≥0 constant for asymptotic purposes. Observe that $n\,\Delta^{3}$ and $n\,\Delta^{\prime ,3}$ are $\tilde{O}\,\left(1/\sqrt{n}\right)$ with probability 1, where the “soft-O” notation $\tilde{O}$ subsumes the polylogarithmic factor from the law of the iterated logarithm. We can now write

$$l_{p}=-\frac{\kappa^{2}}{2\,\lambda }+\tilde{O}\,\left(1/\sqrt{n}\right).$$

Fix the level a and thereby also α=|log⁡(a)|. Define ${\hat{\sigma }}^{\prime }=\sqrt{{\hat{\theta }}_{m}^{\prime }\,\left(1-{\hat{\theta }}_{m}^{\prime }\right)/\left(1-\lambda \right)\,n}$, and let $\varphi^{\prime }={\hat{\theta }}_{m}^{\prime }-\gamma^{\prime }\,{\hat{\sigma }}^{\prime }$ be the smallest solution of $-\log\left(Q_{\lambda }\,\left(𝐛|\varphi^{\prime }\right)\right)=\alpha$. Because

$$-\log\left(Q_{\lambda }\,\left(𝐛|\varphi^{\prime }\right)\right)=-\log\left(P_{\mathrm{CH},\left(1-\lambda \right)\,n}\,\left({\hat{\theta }}_{m}^{\prime }|\varphi^{\prime }\right)\right)+l_{p},$$

we can estimate $\gamma^{\prime }$ as $\gamma^{\prime }=\gamma_{a^{\prime },\left(1-\lambda \right)\,n}\,\left({\hat{\theta }}_{m}^{\prime };P_{\mathrm{CH}}\right)=\sqrt{2\,\left(\alpha -l_{p}\right)}+O\,\left(\alpha /\sqrt{n}\right)$ with $a^{\prime }=e^{-\left(\alpha -l_{p}\right)}$. Here, the subscript (1-λ)⁢n of $\gamma_{a^{\prime }}$ makes the previously implicit number of trials explicit.

To finish our comparison, we express the endpoint $\varphi^{\prime }$ relative to $\hat{\theta }$. For this, we write

$$\varphi^{\prime }={\hat{\theta }}_{m}^{\prime }-\gamma^{\prime }\,{\hat{\sigma }}^{\prime }$$
$$\varphi^{\prime }$$
$$={\hat{\theta }}_{m}^{\prime }-\gamma^{\prime }\,{\hat{\sigma }}^{\prime }$$

$$=\hat{\theta }+\left({\hat{\theta }}_{m}^{\prime }-\hat{\theta }\right)-\gamma^{\prime }\,\hat{\sigma }\,\sqrt{\frac{{\hat{\theta }}_{m}^{\prime }\,\left(1-{\hat{\theta }}_{m}^{\prime }\right)}{\left(1-\lambda \right)\,\hat{\theta }\,\left(1-\hat{\theta }\right)}}$$
$$=\hat{\theta }+\left({\hat{\theta }}_{m}^{\prime }-\hat{\theta }\right)-\gamma^{\prime }\,\hat{\sigma }\,\sqrt{\frac{{\hat{\theta }}_{m}^{\prime }\,\left(1-{\hat{\theta }}_{m}^{\prime }\right)}{\left(1-\lambda \right)\,\hat{\theta }\,\left(1-\hat{\theta }\right)}}$$

$$=\hat{\theta }+\left({\hat{\theta }}_{m}^{\prime }-\hat{\theta }\right)-\frac{\gamma^{\prime }}{\sqrt{1-\lambda }}\,\hat{\sigma }\,\left(1+O\,\left(\left|\hat{\theta }-{\hat{\theta }}_{m}^{\prime }\right|\right)\right).$$
$$=\hat{\theta }+\left({\hat{\theta }}_{m}^{\prime }-\hat{\theta }\right)-\frac{\gamma^{\prime }}{\sqrt{1-\lambda }}\,\hat{\sigma }\,\left(1+O\,\left(\left|\hat{\theta }-{\hat{\theta }}_{m}^{\prime }\right|\right)\right).$$

We have ${\hat{\theta }}_{m}^{\prime }-\hat{\theta }=\lambda \,\left({\hat{\theta }}_{m}^{\prime }-{\hat{\theta }}_{m}\right)=\lambda \,\left(\delta^{\prime }-\delta \right)$, and we are considering the case $\lambda \,\left|\delta^{\prime }-\delta \right|=\kappa \,\sqrt{\lambda \,\theta \,\left(1-\theta \right)/\left(n\,\left(1-\lambda \right)\right)}$, so

$$\varphi^{\prime }=\hat{\theta }-\frac{\gamma^{\prime }}{\sqrt{1-\lambda }}\,\hat{\sigma }\,\left(1+O\,\left(1/\sqrt{n}\right)\right).$$

We can therefore identify

$$\gamma_{a}\,\left(\hat{\theta }|P_{\lambda }\right)=\frac{\gamma^{\prime }}{\sqrt{1-\lambda }}\,\left(1+O\,\left(1/\sqrt{n}\right)\right)$$
$$\gamma_{a}\,\left(\hat{\theta }|P_{\lambda }\right)$$
$$=\frac{\gamma^{\prime }}{\sqrt{1-\lambda }}\,\left(1+O\,\left(1/\sqrt{n}\right)\right)$$

$$=\frac{\sqrt{2\,\left(\alpha +\kappa^{2}/\left(2\,\lambda \right)+\tilde{O}\,\left(1/\sqrt{n}\right)\right)}+O\,\left(\alpha /\sqrt{n}\right)}{\sqrt{1-\lambda }}\,\left(1+O\,\left(1/\sqrt{n}\right)\right)$$
$$=\frac{\sqrt{2\,\left(\alpha +\kappa^{2}/\left(2\,\lambda \right)+\tilde{O}\,\left(1/\sqrt{n}\right)\right)}+O\,\left(\alpha /\sqrt{n}\right)}{\sqrt{1-\lambda }}\,\left(1+O\,\left(1/\sqrt{n}\right)\right)$$

$$=\frac{\sqrt{2\,\left(\alpha +\kappa^{2}/\left(2\,\lambda \right)\right)}}{\sqrt{1-\lambda }}+\tilde{O}\,\left(\alpha /\sqrt{n}\right),$$
$$=\frac{\sqrt{2\,\left(\alpha +\kappa^{2}/\left(2\,\lambda \right)\right)}}{\sqrt{1-\lambda }}+\tilde{O}\,\left(\alpha /\sqrt{n}\right),$$

which compares as promised to $\gamma_{a}\,\left(\hat{\theta };P_{\mathrm{CH}}\right)=\sqrt{2\,\alpha }+O\,\left(\alpha /\sqrt{n}\right)$.

## Conclusion

It is clear that for the specific problem of one-sided hypothesis testing and confidence intervals for Bernoulli RVs, it is always preferable to use the exact test in the ideal case, where the trials are i.i.d. For general nulls, exact tests are typically not available, so approximations are used. The approximations often do not take into account failure of underlying distributional assumptions. The approximation errors can be large at high significance. Thus trustworthy alternatives such as those based on large deviation bounds or test supermartingales are desirable. Our goal here is not to suggest that these alternatives are better for the example of Bernoulli RVs, but to determine the gap between them and an exact test, in a case where an exact test is known and all tests are readily calculable. The suggestion is that for high significance applications, the gaps are relatively small on the relevant logarithmic scale. For p-values, they are within what is expected from experiment-to-experiment variation, even for moderate significances. For confidence intervals, the increase in size is bounded by a constant if the number of trials is known ahead of time, but there is a slowly growing cost with number of trials if we allow for arbitrary stopping-rules.

## Appendix

### Preliminaries

Notation and definitions are as introduced in the text. The p-value bounds obtained by the three tests investigated are denoted by $P_{X}$ for the exact, $P_{\mathrm{CH}}$ for the Chernoff-Hoeffding, and $P_{\mathrm{PBR}}$ for the PBR test. They depend on n, φ and $\hat{\Theta }$. For reference, here are the definitions again.

PX⁢(Θ^|φ,n)=∑i=n⁢Θ^nφi⁢(1-φ)n-i⁢(ni),
$$P_{X}\,\left(\hat{\Theta }|\varphi ,n\right)$$
=∑i=n⁢Θ^nφi⁢(1-φ)n-i⁢(ni),

$$P_{\mathrm{CH}}\,\left(\hat{\Theta }|\varphi \right)=\left\{ \begin{array}{cc} {\left(\frac{\varphi }{\hat{\Theta }}\right)}^{n\,\hat{\Theta }}\,{\left(\frac{1-\varphi }{1-\hat{\Theta }}\right)}^{n\,\left(1-\hat{\Theta }\right)} & \text{if }\hat{\Theta }\geq \varphi \text{,}\\ 1 & \text{otherwise.} \end{array}\right.$$
$$P_{\mathrm{CH}}\,\left(\hat{\Theta }|\varphi \right)$$
$$=\left\{ \begin{array}{cc} {\left(\frac{\varphi }{\hat{\Theta }}\right)}^{n\,\hat{\Theta }}\,{\left(\frac{1-\varphi }{1-\hat{\Theta }}\right)}^{n\,\left(1-\hat{\Theta }\right)} & \text{if }\hat{\Theta }\geq \varphi \text{,}\\ 1 & \text{otherwise.} \end{array}\right.$$

PPBR⁢(Θ^|φ)={φn⁢Θ^⁢(1-φ)n⁢(1-Θ^)⁢(n+1)⁢(nn⁢Θ^)if Θ^≥φ,Θ^n⁢Θ^⁢(1-Θ^)n⁢(1-Θ^)⁢(n+1)⁢(nn⁢Θ^)otherwise.
$$P_{\mathrm{PBR}}\,\left(\hat{\Theta }|\varphi \right)$$
={φn⁢Θ^⁢(1-φ)n⁢(1-Θ^)⁢(n+1)⁢(nn⁢Θ^)if Θ^≥φ,Θ^n⁢Θ^⁢(1-Θ^)n⁢(1-Θ^)⁢(n+1)⁢(nn⁢Θ^)otherwise.

The gain per trial for a p-value bound $P_{n}$ is $G_{n}\,\left(P_{n}\right)=-\log\left(P_{n}\right)/n$. The values of φ, $\hat{\theta }$ and θ are usually constrained. Unless otherwise stated, we assume that $0<\varphi ,\hat{\theta },\theta <1$ and n≥1.

Most of this appendix is dedicated to obtaining upper and lower bounds on log⁡(p)-values and lower bounds on endpoints of confidence intervals. We make sure that the upper and lower bounds differ by quantities that converge to zero as n grows. Their differences are O⁢(1/n) for log⁡(p)-values and $O\,\left(1/\sqrt{n}\right)$ for confidence lower bounds. We generally aim for simplicity when expressing these bounds, so we do not obtain tight constants.

### Closed-Form Expression for $P_{\mathrm{PBR}}$

**.**

*Define*

$${\tilde{\Theta }}_{k}=\frac{1}{k+2}\,\left(S_{k}+1\right),$$
$${\tilde{\Theta }}_{k}$$
$$=\frac{1}{k+2}\,\left(S_{k}+1\right),$$

$$F_{k+1}={\left(\frac{{\tilde{\Theta }}_{k}}{\varphi }\right)}^{B_{k+1}}\,{\left(\frac{1-{\tilde{\Theta }}_{k}}{1-\varphi }\right)}^{1-B_{k+1}}.$$
$$F_{k+1}$$
$$={\left(\frac{{\tilde{\Theta }}_{k}}{\varphi }\right)}^{B_{k+1}}\,{\left(\frac{1-{\tilde{\Theta }}_{k}}{1-\varphi }\right)}^{1-B_{k+1}}.$$

*Then*

1∏k=1nFk=φn⁢Θ^⁢(1-φ)n⁢(1-Θ^)⁢(n+1)⁢(nn⁢Θ^).

**Proof.**

The proof proceeds by induction. Write $P_{k}$ for the right-hand side of Eq. 50. For n=0, $P_{0}=1$, and the left-hand side of Eq. 50 evaluates to 1 as required, with the usual convention that the empty product evaluates to 1.

Now suppose that Eq. 50 holds at trial n=k. For n=k+1 we can use $\left(k+1\right)\,{\hat{\Theta }}_{k+1}=S_{k+1}=S_{k}+B_{k+1}$. We expand the binomial expression to rewrite the right-hand side as

Pk+1=φk⁢Θ^k+Bk+1⁢(1-φ)k⁢(1-Θ^k)+(1-Bk+1)⁢(k+2)⁢(k+1k⁢Θ^k+Bk+1)
$$P_{k+1}$$
=φk⁢Θ^k+Bk+1⁢(1-φ)k⁢(1-Θ^k)+(1-Bk+1)⁢(k+2)⁢(k+1k⁢Θ^k+Bk+1)

=φk⁢Θ^k⁢(1-φ)k⁢(1-Θ^k)⁢(k+1)⁢(kk⁢Θ^k)
=φk⁢Θ^k⁢(1-φ)k⁢(1-Θ^k)⁢(k+1)⁢(kk⁢Θ^k)

$$\cdot \varphi^{B_{k+1}}\,{\left(1-\varphi \right)}^{1-B_{k+1}}\,\left(k+2\right)\,{\left(k-k\,{\hat{\Theta }}_{k}+1\right)}^{-\left(1-B_{k+1}\right)}\,{\left(k\,{\hat{\Theta }}_{k}+1\right)}^{-B_{k+1}}.$$
$$\cdot \varphi^{B_{k+1}}\,{\left(1-\varphi \right)}^{1-B_{k+1}}\,\left(k+2\right)\,{\left(k-k\,{\hat{\Theta }}_{k}+1\right)}^{-\left(1-B_{k+1}\right)}\,{\left(k\,{\hat{\Theta }}_{k}+1\right)}^{-B_{k+1}}.$$

Since ${\tilde{\Theta }}_{k}=\left(S_{k}+1\right)/\left(k+2\right)=\left(k\,{\hat{\Theta }}_{k}+1\right)/\left(k+2\right)$ and $1-{\tilde{\Theta }}_{k}=\left(k-S_{k}+1\right)/\left(k+2\right)=\left(k-k\,{\hat{\Theta }}_{k}+1\right)/\left(k+2\right)$, the identity simplifies to

$$P_{k+1}=P_{k}\cdot \frac{1}{F_{k+1}},$$

thus establishing the induction step. ∎

The expression in Eq. 50 can be seen as the inverse of a positive martingale for ${ℋ}_{0}=\left\{\nu_{\varphi }\right\}$ determined by $S_{n}$. The complete family of such martingales was obtained by Ville [13], Chapter 5, Sec. 3, Eq. 21. Ours is obtained from Ville’s with d⁢F⁢(t)=d⁢t as the probability measure.

### Log-p-Value Approximations

We use $-\log\left(P_{\mathrm{CH},n}\,\left(t|\varphi \right)\right)=n\,\mathrm{KL}\,\left(t|\varphi \right)$ as our reference value. According to Theorem 1, the log⁡(p)-values are ordered according to $-\log\left(P_{\mathrm{PBR}}\right)\leq -\log\left(P_{\mathrm{CH}}\right)\leq -\log\left(P_{X}\right)$. To express the asymptotic differences between the log⁡(p)-values, we use auxiliary functions. The first is

Hn⁢(t)=-log⁡(tn⁢t⁢(1-t)n⁢(1-t)⁢(nn⁢t)⁢n+1)
$$H_{n}\,\left(t\right)$$
=-log⁡(tn⁢t⁢(1-t)n⁢(1-t)⁢(nn⁢t)⁢n+1)

=-n⁢t⁢log⁡(t)-n⁢(1-t)⁢log⁡(1-t)-log⁡(nn⁢t)-12⁢log⁡(n+1).
=-n⁢t⁢log⁡(t)-n⁢(1-t)⁢log⁡(1-t)-log⁡(nn⁢t)-12⁢log⁡(n+1).

The first two terms of this expression can be recognized as the Shannon entropy of n independent random bits, each with probability t for bit value 1. For t∈[1/n,1-1/n] and with Stirling’s approximation $\sqrt{2\,\pi \,n}\,{\left(n/e\right)}^{n}\,e^{1/\left(12\,n+1\right)}\leq n!\leq \sqrt{2\,\pi \,n}\,{\left(n/e\right)}^{n}\,e^{1/\left(12\,n\right)}$ applied to the binomial coefficient, we get

log⁡(nn⁢t)=log⁡(n!(t⁢n)!⁢((1-t)⁢n)!)
log⁡(nn⁢t)
$$=\log\left(\frac{n!}{\left(t\,n\right)!\,\left(\left(1-t\right)\,n\right)!}\right)$$

$$\in \log\left(\frac{\sqrt{2\,\pi \,n}}{\sqrt{2\,\pi \,t\,n}\,\sqrt{2\,\pi \,\left(1-t\right)\,n}}\right)+\log\left(\frac{{\left(n/e\right)}^{n}}{{\left(t\,n/e\right)}^{t\,n}\,{\left(\left(1-t\right)\,n/e\right)}^{\left(1-t\right)\,n}}\right)$$
$$\in \log\left(\frac{\sqrt{2\,\pi \,n}}{\sqrt{2\,\pi \,t\,n}\,\sqrt{2\,\pi \,\left(1-t\right)\,n}}\right)+\log\left(\frac{{\left(n/e\right)}^{n}}{{\left(t\,n/e\right)}^{t\,n}\,{\left(\left(1-t\right)\,n/e\right)}^{\left(1-t\right)\,n}}\right)$$

$$+\left[\frac{1}{12\,n+1},\frac{1}{12\,n}\right]+\left[-\frac{1}{12\,t\,n}-\frac{1}{12\,\left(1-t\right)\,n},-\frac{1}{12\,t\,n+1}-\frac{1}{12\,\left(1-t\right)\,n+1}\right]$$
$$+\left[\frac{1}{12\,n+1},\frac{1}{12\,n}\right]+\left[-\frac{1}{12\,t\,n}-\frac{1}{12\,\left(1-t\right)\,n},-\frac{1}{12\,t\,n+1}-\frac{1}{12\,\left(1-t\right)\,n+1}\right]$$

$$=-\frac{1}{2}\,\log\left(2\,\pi \,t\,\left(1-t\right)\right)-\frac{1}{2}\,\log\left(n\right)-t\,n\,\log\left(t\right)-\left(1-t\right)\,n\,\log\left(1-t\right)$$
$$=-\frac{1}{2}\,\log\left(2\,\pi \,t\,\left(1-t\right)\right)-\frac{1}{2}\,\log\left(n\right)-t\,n\,\log\left(t\right)-\left(1-t\right)\,n\,\log\left(1-t\right)$$

$$+\left[\frac{1}{12\,n+1}-\frac{1}{12\,t\,\left(1-t\right)\,n},\frac{1}{12\,n}-\frac{12\,n+2}{\left(12\,t\,n+1\right)\,\left(12\,\left(1-t\right)\,n+1\right)}\right].$$
$$+\left[\frac{1}{12\,n+1}-\frac{1}{12\,t\,\left(1-t\right)\,n},\frac{1}{12\,n}-\frac{12\,n+2}{\left(12\,t\,n+1\right)\,\left(12\,\left(1-t\right)\,n+1\right)}\right].$$

We can increase the interval to simplify the bounds while preserving convergence for large n. For the lower bound, we use -1/(12⁢t⁢(1-t)⁢n). For the upper bound, note that (12⁢t⁢n+1)⁢(12⁢(1-t)⁢n+1)is maximized at t=1/2. We can therefore increase the upper bound according to

$$\frac{1}{12\,n}-\frac{12\,n+2}{\left(12\,t\,n+1\right)\,\left(12\,\left(1-t\right)\,n+1\right)}\leq \frac{1}{12\,n}-\frac{2}{6\,n+1}\leq 0$$

for n≥1. From this we obtain the interval expression

$$H_{n}\,\left(t\right)\in \frac{1}{2}\,\log\left(2\,\pi \,t\,\left(1-t\right)\right)-\frac{1}{2}\,\log\left(1+1/n\right)+\left[0,\frac{1}{12\,n\,t\,\left(1-t\right)}\right],$$
$$H_{n}\,\left(t\right)$$
$$\in \frac{1}{2}\,\log\left(2\,\pi \,t\,\left(1-t\right)\right)-\frac{1}{2}\,\log\left(1+1/n\right)+\left[0,\frac{1}{12\,n\,t\,\left(1-t\right)}\right],$$

valid for t∈[1/n,1-1/n]. The boundary values of $H_{n}$ at t=0 and t=1 are -log⁡(n+1)/2.

The next auxiliary function is

$$Y\,\left(t\right)=\frac{1}{e^{-t^{2}/2}}\,\int_{t}^{\infty }e^{-s^{2}/2}\,𝑑s\in \left(\frac{t}{1+t^{2}},\frac{1}{t}\right)\,\text{ for }\,t>0,$$

where the bounds are from Ref. [24]. See this reference for a summary of all properties of Y mentioned here, or Ref. [25] for more details. The function Y is related to the tail of the standard normal distribution, the Q-function, by $Q\,\left(t\right)=e^{-t^{2}/2}\,Y\,\left(t\right)/\sqrt{2\,\pi }$. The function Y is monotonically decreasing, convex, $Y\,\left(0\right)=\sqrt{\pi /2}$, and it satisfies the differential equation $\frac{d}{d\,t}\,Y\,\left(t\right)=t\,Y\,\left(t\right)-1$. We make use of the following bounds involving Y:

$$-\log t\,Y\,\left(t\right)\in \left[0,\frac{1}{t^{2}}\right].$$

The lower bound comes from the upper bound 1/t for Y⁢(t). The upper bound is from the lower bound $t/\left(1+t^{2}\right)$ for Y⁢(t). Specifically, we compute $-\log\left(Y\,\left(t\right)\right)\leq -\log\left(t/\left(1+t^{2}\right)\right)=\log\left(t\right)+\log\left(1+1/t^{2}\right)\leq \log\left(t\right)+1/t^{2}$.

With these definitions, we can express the log⁡(p)-values in terms of their difference from $-\log\left(P_{\mathrm{CH}}\right)$.

**.**

*For 0<φ≤t<1,*

$$-\log\left(P_{\mathrm{PBR},n}\,\left(t|\varphi \right)\right)=-\log\left(P_{\mathrm{CH},n}\,\left(t|\varphi \right)\right)-\frac{1}{2}\,\log\left(n+1\right)+H_{n}\,\left(t\right)$$
$$-\log\left(P_{\mathrm{PBR},n}\,\left(t|\varphi \right)\right)$$
$$=-\log\left(P_{\mathrm{CH},n}\,\left(t|\varphi \right)\right)-\frac{1}{2}\,\log\left(n+1\right)+H_{n}\,\left(t\right)$$

$$\in -\log\left(P_{\mathrm{CH},n}\,\left(t|\varphi \right)\right)-\frac{1}{2}\,\log\left(n+1\right)+\frac{1}{2}\,\log\left(2\,\pi \,t\,\left(1-t\right)\right)-\frac{1}{2}\,\log\left(1+1/n\right)$$
$$\in -\log\left(P_{\mathrm{CH},n}\,\left(t|\varphi \right)\right)-\frac{1}{2}\,\log\left(n+1\right)+\frac{1}{2}\,\log\left(2\,\pi \,t\,\left(1-t\right)\right)-\frac{1}{2}\,\log\left(1+1/n\right)$$

$$+\left[0,\frac{1}{12\,n\,t\,\left(1-t\right)},\right]$$
$$+\left[0,\frac{1}{12\,n\,t\,\left(1-t\right)},\right]$$

**Proof.**

The theorem is obtained by substituting definitions and then applying the bounds of Eq. 56 on $H_{n}\,\left(t\right)$. Here are the details.

-log⁡(PPBR,n⁢(t|φ))=-log⁡(φn⁢t⁢(1-φ)n⁢(1-t)⁢(n+1)⁢(nn⁢t))
$$-\log\left(P_{\mathrm{PBR},n}\,\left(t|\varphi \right)\right)$$
=-log⁡(φn⁢t⁢(1-φ)n⁢(1-t)⁢(n+1)⁢(nn⁢t))

$$=-\log\left({\left(\frac{\varphi }{t}\right)}^{n\,t}\,{\left(\frac{1-\varphi }{1-t}\right)}^{n\,\left(1-t\right)}\right)$$
$$=-\log\left({\left(\frac{\varphi }{t}\right)}^{n\,t}\,{\left(\frac{1-\varphi }{1-t}\right)}^{n\,\left(1-t\right)}\right)$$

-log⁡(tn⁢t⁢(1-t)n(1-t))⁢(n+1)⁢(nn⁢t))
-log⁡(tn⁢t⁢(1-t)n(1-t))⁢(n+1)⁢(nn⁢t))

$$=-\log\left(P_{\mathrm{CH},n}\,\left(t|\varphi \right)\right)-\frac{1}{2}\,\log\left(n+1\right)$$
$$=-\log\left(P_{\mathrm{CH},n}\,\left(t|\varphi \right)\right)-\frac{1}{2}\,\log\left(n+1\right)$$

-log⁡(tn⁢t⁢(1-t)n(1-t))⁢n+1⁢(nn⁢t))
-log⁡(tn⁢t⁢(1-t)n(1-t))⁢n+1⁢(nn⁢t))

$$=-\log\left(P_{\mathrm{CH},n}\,\left(t|\varphi \right)\right)-\frac{1}{2}\,\log\left(n+1\right)+H_{n}\,\left(t\right).$$
$$=-\log\left(P_{\mathrm{CH},n}\,\left(t|\varphi \right)\right)-\frac{1}{2}\,\log\left(n+1\right)+H_{n}\,\left(t\right).$$

It remains to substitute the interval expression for $H_{n}\,\left(t\right)$. ∎

**.**

*Define*

$${\mathrm{lE}}_{n}\,\left(t|\varphi \right)=\min\left(\left(t-\varphi \right)\,\sqrt{\frac{\pi \,n}{8\,\varphi \,\left(1-\varphi \right)}},1\right).$$

*Then for 0<φ<t<1,*

$$-\log\left(P_{X,n}\,\left(t|\varphi \right)\right)\in -\log\left(P_{\mathrm{PBR},n}\,\left(t|\varphi \right)\right)+\log\left(n+1\right)-\log\left(t\,\sqrt{\frac{\left(1-\varphi \right)}{\varphi }}\right)$$
$$-\log\left(P_{X,n}\,\left(t|\varphi \right)\right)$$
$$\in -\log\left(P_{\mathrm{PBR},n}\,\left(t|\varphi \right)\right)+\log\left(n+1\right)-\log\left(t\,\sqrt{\frac{\left(1-\varphi \right)}{\varphi }}\right)$$

$$-\log\left(\sqrt{n}\,Y\,\left(\sqrt{\frac{n}{\varphi \,\left(1-\varphi \right)}}\,\left(t-\varphi \right)\right)\right)+\left[-\frac{{\mathrm{lE}}_{n}\,\left(t|\varphi \right)}{n\,\left(t-\varphi \right)},0\right],$$
$$-\log\left(\sqrt{n}\,Y\,\left(\sqrt{\frac{n}{\varphi \,\left(1-\varphi \right)}}\,\left(t-\varphi \right)\right)\right)+\left[-\frac{{\mathrm{lE}}_{n}\,\left(t|\varphi \right)}{n\,\left(t-\varphi \right)},0\right],$$

$$-\log\left(P_{X,n}\,\left(t|\varphi \right)\right)\in -\log\left(P_{\mathrm{CH},n}\,\left(t|\varphi \right)\right)+\frac{1}{2}\,\log\left(n\right)-\log\left(\sqrt{\frac{t\,\left(1-\varphi \right)}{2\,\pi \,\left(1-t\right)\,\varphi }}\right)$$
$$-\log\left(P_{X,n}\,\left(t|\varphi \right)\right)$$
$$\in -\log\left(P_{\mathrm{CH},n}\,\left(t|\varphi \right)\right)+\frac{1}{2}\,\log\left(n\right)-\log\left(\sqrt{\frac{t\,\left(1-\varphi \right)}{2\,\pi \,\left(1-t\right)\,\varphi }}\right)$$

$$-\log\left(\sqrt{n}\,Y\,\left(\sqrt{\frac{n}{\varphi \,\left(1-\varphi \right)}}\,\left(t-\varphi \right)\right)\right)+\left[-\frac{{\mathrm{lE}}_{n}\,\left(t|\varphi \right)}{n\,\left(t-\varphi \right)},\frac{1}{12\,n\,t\,\left(1-t\right)}\right].$$
$$-\log\left(\sqrt{n}\,Y\,\left(\sqrt{\frac{n}{\varphi \,\left(1-\varphi \right)}}\,\left(t-\varphi \right)\right)\right)+\left[-\frac{{\mathrm{lE}}_{n}\,\left(t|\varphi \right)}{n\,\left(t-\varphi \right)},\frac{1}{12\,n\,t\,\left(1-t\right)}\right].$$

Observe that ${\mathrm{lE}}_{n}\,\left(t|\varphi \right)$ is O⁢(1) with respect to n for t>φ constant. The first term in the defining minimum is smaller than 1 only for φ within less than one standard deviation (which is $O\,\left(1/\sqrt{n}\right)$) of t. It is defined so that the primary dependence on the parameters is visible in the interval bounds.

**Proof.**

For approximating $P_{X}$, we apply Theorem 2 of Ref. [24] with the following sequence of substitutions, the first four of which expand the definitions in the reference:

$$B\,\left(k;n,p\right)\leftarrow \sum_{j=k}^{n}b\,\left(j;n,p\right),$$
B⁢(k;n,p)
$$\leftarrow \sum_{j=k}^{n}b\,\left(j;n,p\right),$$

b⁢(k-1;n-1,p)←(n-1k-1)⁢pk-1⁢(1-p)n-k,
b⁢(k-1;n-1,p)
←(n-1k-1)⁢pk-1⁢(1-p)n-k,

x←(k-p⁢n)/σ,
x
←(k-p⁢n)/σ,

$$\sigma \leftarrow \sqrt{n\,p\,\left(1-p\right)},$$
σ
$$\leftarrow \sqrt{n\,p\,\left(1-p\right)},$$

p←φ,
p
←φ,

k←n⁢t.
k
←n⁢t.

With the given substitutions and Y⁢(t) as defined by Eq. 57, we obtain for t≥φ,

-log⁡(PX)∈-log⁡(n⁢φ⁢(1-φ)⁢φn⁢t-1⁢(1-φ)n⁢(1-t)⁢(n-1n⁢t-1))
$$-\log\left(P_{X}\right)$$
∈-log⁡(n⁢φ⁢(1-φ)⁢φn⁢t-1⁢(1-φ)n⁢(1-t)⁢(n-1n⁢t-1))

$$-\log\left(Y\,\left(\frac{\sqrt{n}\,\left(t-\varphi \right)}{\sqrt{\varphi \,\left(1-\varphi \right)}}\right)\right)+\left[-\frac{{\mathrm{lE}}_{n}\,\left(t|\varphi \right)}{n\,\left(t-\varphi \right)},0\right]$$
$$-\log\left(Y\,\left(\frac{\sqrt{n}\,\left(t-\varphi \right)}{\sqrt{\varphi \,\left(1-\varphi \right)}}\right)\right)+\left[-\frac{{\mathrm{lE}}_{n}\,\left(t|\varphi \right)}{n\,\left(t-\varphi \right)},0\right]$$

=-log⁡(φn⁢t⁢(1-φ)n⁢(1-t)⁢(n+1)⁢(nn⁢t))-log⁡(n⁢t⁢n⁢φ⁢(1-φ)φ⁢n⁢(n+1))
=-log⁡(φn⁢t⁢(1-φ)n⁢(1-t)⁢(n+1)⁢(nn⁢t))-log⁡(n⁢t⁢n⁢φ⁢(1-φ)φ⁢n⁢(n+1))

$$-\log\left(Y\,\left(\frac{\sqrt{n}\,\left(t-\varphi \right)}{\sqrt{\varphi \,\left(1-\varphi \right)}}\right)\right)+\left[-\frac{{\mathrm{lE}}_{n}\,\left(t|\varphi \right)}{n\,\left(t-\varphi \right)},0\right]$$
$$-\log\left(Y\,\left(\frac{\sqrt{n}\,\left(t-\varphi \right)}{\sqrt{\varphi \,\left(1-\varphi \right)}}\right)\right)+\left[-\frac{{\mathrm{lE}}_{n}\,\left(t|\varphi \right)}{n\,\left(t-\varphi \right)},0\right]$$

$$=-\log\left(P_{\mathrm{PBR}}\right)+\log\left(n+1\right)-\log\left(t\,\sqrt{\frac{\left(1-\varphi \right)}{\varphi }}\right)$$
$$=-\log\left(P_{\mathrm{PBR}}\right)+\log\left(n+1\right)-\log\left(t\,\sqrt{\frac{\left(1-\varphi \right)}{\varphi }}\right)$$

$$-\log\left(\sqrt{n}\,Y\,\left(\frac{\sqrt{n}\,\left(t-\varphi \right)}{\sqrt{\varphi \,\left(1-\varphi \right)}}\right)\right)+\left[-\frac{{\mathrm{lE}}_{n}\,\left(t|\varphi \right)}{n\,\left(t-\varphi \right)},0\right].$$
$$-\log\left(\sqrt{n}\,Y\,\left(\frac{\sqrt{n}\,\left(t-\varphi \right)}{\sqrt{\varphi \,\left(1-\varphi \right)}}\right)\right)+\left[-\frac{{\mathrm{lE}}_{n}\,\left(t|\varphi \right)}{n\,\left(t-\varphi \right)},0\right].$$

The second identity of the theorem follows by substituting the expression from Theorem 6. ∎

We can eliminate the function Y from the expressions by applying the bounds from Eq. 58.

**.**

*With the assumptions of Theorem 7, *

$$-\log\left(P_{X,n}\,\left(t|\varphi \right)\right)\in -\log\left(P_{\mathrm{CH},n}\,\left(t|\varphi \right)\right)+\frac{1}{2}\,\log\left(n\right)-\log\left(\frac{1-\varphi }{t-\varphi }\,\sqrt{\frac{t}{2\,\pi \,\left(1-t\right)}}\right)$$
$$-\log\left(P_{X,n}\,\left(t|\varphi \right)\right)$$
$$\in -\log\left(P_{\mathrm{CH},n}\,\left(t|\varphi \right)\right)+\frac{1}{2}\,\log\left(n\right)-\log\left(\frac{1-\varphi }{t-\varphi }\,\sqrt{\frac{t}{2\,\pi \,\left(1-t\right)}}\right)$$

$$+\left[-\frac{{\mathrm{lE}}_{n}\,\left(t|\varphi \right)}{n\,\left(t-\varphi \right)},\frac{\varphi \,\left(1-\varphi \right)}{{\left(t-\varphi \right)}^{2}\,n}+\frac{1}{12\,n\,t\,\left(1-t\right)}\right].$$
$$+\left[-\frac{{\mathrm{lE}}_{n}\,\left(t|\varphi \right)}{n\,\left(t-\varphi \right)},\frac{\varphi \,\left(1-\varphi \right)}{{\left(t-\varphi \right)}^{2}\,n}+\frac{1}{12\,n\,t\,\left(1-t\right)}\right].$$

**Proof.**

Define $c=\left(t-\varphi \right)/\sqrt{\varphi \,\left(1-\varphi \right)}$. In view of Eq. 58, we have

$$-\log\left(\sqrt{n}\,Y\,\left(\sqrt{\frac{n}{\varphi \,\left(1-\varphi \right)}}\,\left(t-\varphi \right)\right)\right)=-\log\left(\sqrt{n}\,Y\,\left(c\,\sqrt{n}\right)\right)$$
$$-\log\left(\sqrt{n}\,Y\,\left(\sqrt{\frac{n}{\varphi \,\left(1-\varphi \right)}}\,\left(t-\varphi \right)\right)\right)$$
$$=-\log\left(\sqrt{n}\,Y\,\left(c\,\sqrt{n}\right)\right)$$

$$=\log\left(c\right)-\log\left(c\,\sqrt{n}\,Y\,\left(c\,\sqrt{n}\right)\right)$$
$$=\log\left(c\right)-\log\left(c\,\sqrt{n}\,Y\,\left(c\,\sqrt{n}\right)\right)$$

$$\in \log\left(c\right)+\left[0,\frac{1}{c^{2}\,n}\right].$$
$$\in \log\left(c\right)+\left[0,\frac{1}{c^{2}\,n}\right].$$

Substituting in Eq. 64 and simplifying the expression gives the desired result. ∎

### Asymptotic Normality of the log⁡(p)-Values and Their Differences

The main tool for establishing the asymptotic distribution of the log⁡(p)-values is the “delta method”. A version sufficient for our purposes is Theorem 1.12 and Corollary 1.1 of Ref. [18]. The notation $X_{n}\overset{𝐷}{\to }N\,\left(\mu ,\sigma^{2}\right)$ means that $X_{n}$ converges in distribution to the normal distribution with mean μ and variance $\sigma^{2}$. By the central limit theorem, ${\hat{\Theta }}_{n}=S_{n}/n$ satisfies $\sqrt{n}\,\left({\hat{\Theta }}_{n}-\theta \right)\overset{𝐷}{\to }N\,\left(0,\theta \,\left(1-\theta \right)\right)$. An application of the delta method therefore yields the next lemma.

**.**

*Let F:R→R be differentiable at θ, with $F^{\prime }\,\left(\theta \right)\neq 0$. Then*

$$\sqrt{n}\,\left(F\,\left({\hat{\Theta }}_{n}\right)-F\,\left(\theta \right)\right)\overset{𝐷}{\to }N\,\left(0,F^{\prime }\,{\left(\theta \right)}^{2}\,\theta \,\left(1-\theta \right)\right)$$

**.**

*For $P=P_{\mathrm{CH}}$, $P=P_{\mathrm{PBR}}$ or $P=P_{X}$, and 0<φ<θ<1 constant, the gain per trial $G_{n}\,\left(P\right)$ converges in distribution according to*

$$\sqrt{n}\,\left(G_{n}\,\left(P\right)-\mathrm{KL}\,\left(\theta |\varphi \right)\right)\overset{𝐷}{\to }N\,\left(0,\sigma_{G}^{2}\right),$$

*with*

$$\sigma_{G}^{2}=\theta \,\left(1-\theta \right)\,{\left(\log\left(\frac{\theta }{1-\theta }\,\frac{1-\varphi }{\varphi }\right)\right)}^{2}.$$

**Proof.**

Consider $P=P_{\mathrm{CH}}$ first. In Lemma 9, define F⁢(x)=KL⁢(x|φ)=x⁢log⁡(x/φ)+(1-x)⁢log⁡((1-x)/(1-φ)) so that $F\,\left({\hat{\Theta }}_{n}\right)=G_{n}\,\left(P_{\mathrm{CH}}\right)$. For the derivative of F at x=θ, we get

$$F^{\prime }\,\left(\theta \right)=\log\left(\frac{\theta }{1-\theta }\,\frac{1-\varphi }{\varphi }\right).$$

The theorem now follows for $P_{\mathrm{CH}}$ by applying Lemma 9.

Theorem 6 and the law of large numbers imply that $\left(-\log\left(P_{\mathrm{PBR}}\right)/\sqrt{n}\right)-\left(-\log\left(P_{\mathrm{CH}}\right)/\sqrt{n}\right)$ converges in probability to 0. Corollary 8 implies the same for $P_{X}$, namely that $\left(-\log\left(P_{X}\right)/\sqrt{n}\right)-\left(-\log\left(P_{\mathrm{CH}}\right)/\sqrt{n}\right)$ converges in probability to 0. In general, if $X_{n}-Y_{n}$ converges in probability to 0 and $Y_{n}\overset{𝐷}{\to }\mu$, then $X_{n}\overset{𝐷}{\to }\mu$, see Ref. [26], Proposition 6.3.3. The statement of the theorem to be proven now follows for $P=P_{\mathrm{PBR}}$ and $P=P_{X}$ by comparison of $\sqrt{n}\,G_{n}\,\left(P_{\mathrm{PBR}}\right)$ and $\sqrt{n}\,G_{n}\,\left(P_{X}\right)$ to $\sqrt{n}\,G_{n}\,\left(P_{\mathrm{CH}}\right)$. ∎

The differences of the log⁡(p)-values have much tighter distributions. They are also asymptotically normal with scaling and variances given in the next theorem. The differences are Ω⁢(log⁡(n)) with standard deviations $O\,\left(1/\sqrt{n}\right)$.

**.**

*Let 0<φ<θ<1 be constant. If θ≠1/2, then $P_{\mathrm{PBR}}/\left(\sqrt{n}\,P_{\mathrm{CH}}\right)$ satisfies*

$$-\sqrt{n}\,\log\left(\frac{\sqrt{2\,\pi \,\theta \,\left(1-\theta \right)}\,P_{\mathrm{PBR}}}{\sqrt{n}\,P_{\mathrm{CH}}}\right)\overset{𝐷}{\to }N\,\left(0,\frac{{\left(1-2\,\theta \right)}^{2}}{4\,\theta \,\left(1-\theta \right)}\right).$$
$$-\sqrt{n}\,\log\left(\frac{\sqrt{2\,\pi \,\theta \,\left(1-\theta \right)}\,P_{\mathrm{PBR}}}{\sqrt{n}\,P_{\mathrm{CH}}}\right)$$
$$\overset{𝐷}{\to }N\,\left(0,\frac{{\left(1-2\,\theta \right)}^{2}}{4\,\theta \,\left(1-\theta \right)}\right).$$

*If φ≠θ⁢(2⁢θ-1), then $\sqrt{n}\,P_{X}/P_{\mathrm{CH}}$ satisfies*

$$-\sqrt{n}\,\log\left(\frac{\theta -\varphi }{1-\varphi }\,\sqrt{\frac{2\,\pi \,\left(1-\theta \right)}{\theta }}\,\frac{\sqrt{n}\,P_{X}}{P_{\mathrm{CH}}}\right)\overset{𝐷}{\to }N\,\left(0,\frac{{\left(\theta \,\left(1-2\,\theta \right)+\varphi \right)}^{2}}{4\,{\left(\theta -\varphi \right)}^{2}\,\theta \,\left(1-\theta \right)}\right),$$
$$-\sqrt{n}\,\log\left(\frac{\theta -\varphi }{1-\varphi }\,\sqrt{\frac{2\,\pi \,\left(1-\theta \right)}{\theta }}\,\frac{\sqrt{n}\,P_{X}}{P_{\mathrm{CH}}}\right)$$
$$\overset{𝐷}{\to }N\,\left(0,\frac{{\left(\theta \,\left(1-2\,\theta \right)+\varphi \right)}^{2}}{4\,{\left(\theta -\varphi \right)}^{2}\,\theta \,\left(1-\theta \right)}\right),$$

**Proof.**

From Theorem 6, Eq. 60 and the law of large numbers, we see that

$$\sqrt{n}\,\left(-\log\left(\frac{P_{\mathrm{PBR}}}{\sqrt{n}\,P_{\mathrm{CH}}}\right)-\log\left(\sqrt{2\,\pi \,\hat{\Theta }\,\left(1-\hat{\Theta }\right)}\right)\right)$$

converges in probability to zero. From Lemma 9 and

$$\frac{d}{d\,x}\,\log\left(x\,\left(1-x\right)\right)/2=\frac{1}{2\,x}-\frac{1}{2\,\left(1-x\right)}=\frac{1-2\,x}{2\,x\,\left(1-x\right)},$$

we conclude

$$\sqrt{n}\,\left(\log\left(\sqrt{2\,\pi \,\hat{\Theta }\,\left(1-\hat{\Theta }\right)}\right)-\log\left(\sqrt{2\,\pi \,\theta \,\left(1-\theta \right)}\right)\right)\overset{𝐷}{\to }N\,\left(0,{\left(\frac{1-2\,\theta }{2\,\theta \,\left(1-\theta \right)}\right)}^{2}\,\theta \,\left(1-\theta \right)\right).$$

Combining the above observations gives Eq. 71.

Similarly, from Corollary 8 and taking note of the definition of ${\mathrm{lE}}_{n}\,\left(t|\varphi \right)$,

$$\sqrt{n}\,\left(-\log\left(\frac{\sqrt{n}\,P_{X}}{P_{\mathrm{CH}}}\right)-\log\left(\frac{\hat{\Theta }-\varphi }{1-\varphi }\,\sqrt{\frac{2\,\pi \,\left(1-\hat{\Theta }\right)}{\hat{\Theta }}}\right)\right)$$

converges in probability to zero. The relevant derivative is

$$\frac{d}{d\,x}\,\left(\log\left(x-\varphi \right)+\log\left(\left(1-x\right)/x\right)/2\right)=\frac{1}{x-\varphi }-\frac{1}{2\,\left(1-x\right)}-\frac{1}{2\,x}=\frac{x\,\left(1-2\,x\right)+\varphi }{2\,\left(x-\varphi \right)\,x\,\left(1-x\right)},$$

from which

$$\sqrt{n}\,\left(\log\left(\frac{\hat{\Theta }-\varphi }{1-\varphi }\,\sqrt{\frac{2\,\pi \,\left(1-\hat{\Theta }\right)}{\hat{\Theta }}}\right)-\log\left(\frac{\theta -\varphi }{1-\varphi }\,\sqrt{\frac{2\,\pi \,\left(1-\hat{\theta }\right)}{\hat{\theta }}}\right)\right)$$
$$\sqrt{n}\,\left(\log\left(\frac{\hat{\Theta }-\varphi }{1-\varphi }\,\sqrt{\frac{2\,\pi \,\left(1-\hat{\Theta }\right)}{\hat{\Theta }}}\right)-\log\left(\frac{\theta -\varphi }{1-\varphi }\,\sqrt{\frac{2\,\pi \,\left(1-\hat{\theta }\right)}{\hat{\theta }}}\right)\right)$$

$$\overset{𝐷}{\to }N\,\left(0,{\left(\frac{\theta \,\left(1-2\,\theta \right)+\varphi }{2\,\left(\theta -\varphi \right)\,\theta \,\left(1-\theta \right)}\right)}^{2}\,\theta \,\left(1-\theta \right)\right),$$
$$\overset{𝐷}{\to }N\,\left(0,{\left(\frac{\theta \,\left(1-2\,\theta \right)+\varphi }{2\,\left(\theta -\varphi \right)\,\theta \,\left(1-\theta \right)}\right)}^{2}\,\theta \,\left(1-\theta \right)\right),$$

and combining the two observations gives Eq. 72. ∎

### Confidence Interval Endpoints

For the one-sided confidence intervals, we need to determine the lower boundaries of acceptance regions, that is the confidence lower bounds. By monotonicity of the p-values in φ, it suffices to solve equations of the form $-\log\left(P\,\left(\hat{\theta },\varphi \right)\right)=\alpha$, where $a=e^{-\alpha }$ is the desired significance level. Here we obtain lower and upper bounds on the solutions φ.

To illuminate the asymptotic behavior of solutions φ of $-\log\left(P\,\left(\hat{\theta },\varphi \right)\right)=\alpha$, we reparametrize the log-p-values so that our scale is set by an empirical standard deviation, namely $\hat{\sigma }=\sqrt{\hat{\theta }\,\left(1-\hat{\theta }\right)/n}$. Thus we express the solution as

$$\varphi \,\left(\gamma ,\hat{\theta }\right)=\hat{\theta }-\hat{\sigma }\,\gamma ,$$

in terms of a scaled deviation down from $\hat{\theta }$. Inverting for γ we get

$$\gamma =\gamma \,\left(\varphi ,\hat{\theta }\right)=\frac{\hat{\theta }-\varphi }{\hat{\sigma }}.$$

**.**

*Let $0<\hat{\theta }<1$ and α>0. Suppose that $\alpha \leq n\,{\hat{\theta }}^{2}\,{\left(1-\hat{\theta }\right)}^{2}/8$. Then there is a solution $\gamma_{\alpha }>0$ of the identity $-\log\left(P_{\mathrm{CH}}\,\left(\hat{\theta },\varphi \,\left(\gamma_{\alpha },\hat{\theta }\right)\right)\right)=\alpha$ satisfying*

$$\gamma_{\alpha }\in \sqrt{2\,\alpha }\,{\left(1+\frac{5}{2}\,\frac{\sqrt{\alpha }}{\sqrt{n\,\hat{\theta }\,\left(1-\hat{\theta }\right)}}\,\left[-1,1\right]\right)}^{-1/2}.$$

The constants in this theorem and elsewhere are chosen for convenience, not for optimality; better constants can be extracted from the proofs. Note that the upper bound on α ensures that the reciprocal square root is bounded away from zero. However, for the relative error to go to zero as n grows requires α=o⁢(n).

**Proof.**

Consider the parametrized bound $\alpha \leq 2\,n\,{\hat{\theta }}^{2}\,{\left(1-\hat{\theta }\right)}^{2}\,{\left(1-a_{1}\right)}^{2}$, where later we set $a_{1}=3/4$ to match the theorem statement. Let $F\,\left(\gamma \right)=-\log\left(P_{\mathrm{CH}}\,\left(\hat{\theta },\varphi \,\left(\gamma ,\hat{\theta }\right)\right)\right)$. F is continuous and monotone increasing. A standard simplification of the Chernoff-Hoeffding bound noted in Ref. [17] is

$$P_{\mathrm{CH}}\leq e^{-2\,n\,{\left(\hat{\theta }-\varphi \right)}^{2}}=e^{-2\,\hat{\theta }\,\left(1-\hat{\theta }\right)\,\gamma^{2}}.$$

For $\varphi =\varphi \,\left(\gamma_{\alpha },\hat{\theta }\right)$ solving the desired equation, we have $\left(\hat{\theta }-\varphi \right)\leq \sqrt{\alpha /\left(2\,n\right)}$ (by monotonicity), which in turn is bounded above according to $\sqrt{\alpha /2\,n}\leq \hat{\theta }\,\left(1-\hat{\theta }\right)\,\left(1-a_{1}\right)\leq \hat{\theta }\,\left(1-a_{1}\right)$, according to our assumed bound. We conclude that $\varphi \geq a_{1}\,\hat{\theta }$. For the solution $\gamma_{\alpha }$, we get $\gamma_{\alpha }\leq \sqrt{\alpha /\left(2\,\hat{\theta }\,\left(1-\hat{\theta }\right)\right)}\leq \sqrt{n\,\hat{\theta }\,\left(1-\hat{\theta }\right)}\,\left(1-a_{1}\right)$.

We now Taylor expand $\mathrm{KL}\,\left(\hat{\theta }|\varphi \right)$ with remainder at third order around $\varphi =\hat{\theta }$. Write $f\,\left(x\right)=\mathrm{KL}\,\left(\hat{\theta }|\hat{\theta }-x\right)$, where we can restrict x according to $\hat{\theta }\geq \hat{\theta }-x=\varphi \geq a_{1}\,\hat{\theta }$. The derivatives of f can be written explicitly as follows:

$$f^{\left(k\right)}\,\left(x\right)=\left(k-1\right)!\,\frac{\hat{\theta }}{{\left(\hat{\theta }-x\right)}^{k}}-{\left(-1\right)}^{k-1}\,\left(k-1\right)!\,\frac{1-\hat{\theta }}{{\left(1-\hat{\theta }+x\right)}^{k}}.$$

We have

$$f^{\left(1\right)}\,\left(0\right)=0,$$
$$f^{\left(1\right)}\,\left(0\right)$$
=0,

$$f^{\left(2\right)}\,\left(0\right)=\frac{1}{\hat{\theta }}+\frac{1}{1-\hat{\theta }}=\frac{1}{\hat{\theta }\,\left(1-\hat{\theta }\right)},$$
$$f^{\left(2\right)}\,\left(0\right)$$
$$=\frac{1}{\hat{\theta }}+\frac{1}{1-\hat{\theta }}=\frac{1}{\hat{\theta }\,\left(1-\hat{\theta }\right)},$$

$$f^{\left(3\right)}\,\left(x\right)=2\,\frac{\hat{\theta }}{{\left(\hat{\theta }-x\right)}^{3}}-2\,\frac{1-\hat{\theta }}{{\left(1-\hat{\theta }+x\right)}^{3}},$$
$$f^{\left(3\right)}\,\left(x\right)$$
$$=2\,\frac{\hat{\theta }}{{\left(\hat{\theta }-x\right)}^{3}}-2\,\frac{1-\hat{\theta }}{{\left(1-\hat{\theta }+x\right)}^{3}},$$

$$f^{\left(3\right)}\,\left(x\right)\leq 2\,\frac{\hat{\theta }}{{\left(\hat{\theta }-x\right)}^{3}}\leq 2\,\frac{\hat{\theta }}{a_{1}^{3}\,{\hat{\theta }}^{3}}=2\,\frac{1}{a_{1}^{3}\,{\hat{\theta }}^{2}},$$
$$f^{\left(3\right)}\,\left(x\right)$$
$$\leq 2\,\frac{\hat{\theta }}{{\left(\hat{\theta }-x\right)}^{3}}\leq 2\,\frac{\hat{\theta }}{a_{1}^{3}\,{\hat{\theta }}^{3}}=2\,\frac{1}{a_{1}^{3}\,{\hat{\theta }}^{2}},$$

$$f^{\left(3\right)}\,\left(x\right)\geq -2\,\frac{1-\hat{\theta }}{{\left(1-\hat{\theta }+x\right)}^{3}}\geq -2\,\frac{1-\hat{\theta }}{{\left(1-\hat{\theta }\right)}^{3}}=-2\,\frac{1}{{\left(1-\hat{\theta }\right)}^{2}},$$
$$f^{\left(3\right)}\,\left(x\right)$$
$$\geq -2\,\frac{1-\hat{\theta }}{{\left(1-\hat{\theta }+x\right)}^{3}}\geq -2\,\frac{1-\hat{\theta }}{{\left(1-\hat{\theta }\right)}^{3}}=-2\,\frac{1}{{\left(1-\hat{\theta }\right)}^{2}},$$

since $0<a_{1}<1$. We use the bounds on $f^{\left(3\right)}\,\left(x\right)$ to bound the remainder in the Taylor expansion, where, to get cleaner expressions, we can decrease $\hat{\theta }$ and $1-\hat{\theta }$ to $\hat{\theta }\,\left(1-\hat{\theta }\right)$ in the denominators.

$$\mathrm{KL}\,\left(\hat{\theta }|\hat{\theta }-x\right)\in \frac{x^{2}}{2\,\hat{\theta }\,\left(1-\hat{\theta }\right)}+\frac{x^{3}}{3\,{\left(\hat{\theta }\,\left(1-\hat{\theta }\right)\right)}^{2}}\,\left[-1,\frac{1}{a_{1}^{3}}\right].$$

Substituting $x=\gamma_{\alpha }\,\sqrt{\hat{\theta }\,\left(1-\hat{\theta }\right)/n}$ gives

$$\alpha =-\log\left(P_{\mathrm{CH}}\,\left(\hat{\theta },\varphi \,\left(\gamma_{\alpha },\hat{\theta }\right)\right)\right)=n\,\mathrm{KL}\,\left(\hat{\theta }|\hat{\theta }-x\right)\in \frac{\gamma_{\alpha }^{2}}{2}\,\left(1+\frac{2\,\gamma_{\alpha }}{3\,\sqrt{n\,\hat{\theta }\,\left(1-\hat{\theta }\right)}}\,\left[-1,\frac{1}{a_{1}^{3}}\right]\right).$$

For $\hat{\theta }\leq 1/2$, $f^{\left(4\right)}\,\left(x\right)$ and $f^{\left(3\right)}\,\left(0\right)$ are non-negative, so we could have taken the lower bound in the interval to be zero for θ≤1/2. For the theorem, we prefer not to separate the cases.

We substitute the bound $\gamma \leq \sqrt{n\,\hat{\theta }\,\left(1-\hat{\theta }\right)}\,\left(1-a_{1}\right)$ for the γ multiplying the interval in Eq. 86 and use the lower bound in the interval for the inequality

$$\alpha \geq \frac{\gamma^{2}}{2}\,\left(1-\frac{2\,\left(1-a_{1}\right)}{3}\right).$$

For the theorem, we have $a_{1}=3/4$, so $1-2\,\left(1-a_{1}\right)/3=5/6$. Inverting the inequality for γ gives $\gamma \leq 2\,\sqrt{3/5}\,\sqrt{\alpha }$. Now substituting this bound on γ for the γ multiplying the interval in Eq. 86 gives

$$\alpha \in \frac{\gamma^{2}}{2}\,\left(1+\frac{4}{\sqrt{15}}\,\frac{\sqrt{\alpha }}{\sqrt{n\,\hat{\theta }\,\left(1-\hat{\theta }\right)}}\,\left[-1,\frac{4^{3}}{3^{3}}\right]\right).$$

By monotonicity of the appropriate operations,

$$\gamma \in \sqrt{2\,\alpha }\,{\left(1+\frac{4}{\sqrt{15}}\,\frac{\sqrt{\alpha }}{\sqrt{n\,\hat{\theta }\,\left(1-\hat{\theta }\right)}}\,\left[-1,\frac{4^{3}}{3^{3}}\right]\right)}^{-1/2}.$$

For the theorem statement, we simplify the bounds with $1\leq 4^{3}/3^{3}$ and $4^{4}/\left(3^{3}\,\sqrt{15}\right)\leq 5/2$. ∎

**.**

*Let $0<\hat{\theta }<1$ and α>0. Define $\Delta =\log\left(n+1\right)/2-H_{n}\,\left(\hat{\theta }\right)$. Suppose that $\alpha +\Delta \leq n\,{\hat{\theta }}^{2}\,{\left(1-\hat{\theta }\right)}^{2}/8$. Then there is a solution $\gamma_{\alpha }>0$ of the identity $-\log\left(P_{\mathrm{PBR}}\,\left(\hat{\theta },\varphi \,\left(\gamma_{\alpha },\hat{\theta }\right)\right)\right)=\alpha$ satisfying*

$$\gamma_{\alpha }\in \sqrt{2\,\left(\alpha +\Delta \right)}\,{\left(1+\frac{5}{2}\,\frac{\sqrt{\alpha +\Delta }}{\sqrt{n\,\hat{\theta }\,\left(1-\hat{\theta }\right)}}\,\left[-1,1\right]\right)}^{-1/2}.$$

**Proof.**

By Theorem 6, $-\log\left(P_{\mathrm{CH}}\right)-\left(-\log\left(P_{\mathrm{PBR}}\right)\right)=\Delta$. If we define $\tilde{\alpha }=\alpha +\Delta$, then solving $-\log\left(P_{\mathrm{PBR}}\right)=\alpha$ is equivalent to solving $-\log\left(P_{\mathrm{CH}}\right)=\tilde{\alpha }$. Since Δ depends only on n and $\hat{\theta }$, $\tilde{\alpha }$ does not depend on γ. We can therefore apply Theorem 12 to get the desired bounds. ∎

**.**

*For x≥0, let $q\,\left(x\right)=-\log\left(e^{-x^{2}/2}\,Y\,\left(x\right)/\sqrt{2\,\pi }\right)=x^{2}/2+\log\left(2\,\pi \right)/2-\log\left(Y\,\left(x\right)\right)$. Suppose that $0<\hat{\theta }<1$, and $\log\left(2\right)<\alpha \leq n\,{\hat{\theta }}^{2}\,{\left(1-\hat{\theta }\right)}^{2}/8$. Then there is a solution $\gamma_{\alpha }$ of the identity $-\log(P_{X}\left(\hat{\theta },\varphi \left(\gamma_{\alpha },\hat{\theta }\right)\right)=\alpha$ satisfying*

$$\gamma_{\alpha }\in \max\left(0,q^{-1}\,\left(\alpha \,\left(1+\frac{64\,\sqrt{\alpha }/\left(15\,\sqrt{15}\right)}{\sqrt{n\,\hat{\theta }\,\left(1-\hat{\theta }\right)}}\,\left[-1,1\right]\right)+\frac{\sqrt{\pi /6}+8\,\sqrt{\alpha }/\sqrt{15}}{\sqrt{n\,\hat{\theta }\,\left(1-\hat{\theta }\right)}}\,\left[-1,1\right]\right)\right)$$
$$\gamma_{\alpha }$$
$$\in \max\left(0,q^{-1}\,\left(\alpha \,\left(1+\frac{64\,\sqrt{\alpha }/\left(15\,\sqrt{15}\right)}{\sqrt{n\,\hat{\theta }\,\left(1-\hat{\theta }\right)}}\,\left[-1,1\right]\right)+\frac{\sqrt{\pi /6}+8\,\sqrt{\alpha }/\sqrt{15}}{\sqrt{n\,\hat{\theta }\,\left(1-\hat{\theta }\right)}}\,\left[-1,1\right]\right)\right)$$

$$\times \left(1+\frac{2\,\sqrt{\alpha }/\sqrt{5}}{\sqrt{n\,\hat{\theta }\,\left(1-\hat{\theta }\right)}}\,\left[-1,1\right]\right),$$
$$\times \left(1+\frac{2\,\sqrt{\alpha }/\sqrt{5}}{\sqrt{n\,\hat{\theta }\,\left(1-\hat{\theta }\right)}}\,\left[-1,1\right]\right),$$

*where we extend $q^{-1}$ to negative values by $q^{-1}\,\left(y\right)=-\infty$ for y≤0 (if necessary) when evaluating this interval expression.*

The function q⁢(x) is the negative logarithm of the Q-function, which is the tail of the standard normal distribution. The lower bound on α in Theorem 14 ensures that there is a solution with $\gamma_{\alpha }>0$, because q⁢(0)=log⁡(2). For reference, the constants multiplying the interval expressions are $64/\left(15\,\sqrt{15}\right)\approx 1.102$, $8/\sqrt{15}\approx 2.066$, $\sqrt{\pi /6}\approx 0.724$, $2/\sqrt{5}\approx 0.894$. Note that in the large n limit, where the $O\,\left(1/\sqrt{n}\right)$ terms are negligible, the value of $\gamma_{\alpha }$ in Theorem 14 corresponds to the $\left(1-e^{-\alpha }\right)$-quantile of the standard normal.

By monotonicity of $q^{-1}$, the explicit bounds in Eq. 90 are obtained by combining the lower or the upper bounds in intervals in the expression. We remark that $q^{-1}$ behaves well with respect to relative error for αlarge enough because of the inequalities

$$q^{-1}\,\left(y\right)/\left(1+q^{-1}\,{\left(y\right)}^{2}\right)\leq \frac{d}{d\,y}\,q^{-1}\,\left(y\right)\leq 1/q^{-1}\,\left(y\right),$$
$$q^{-1}\,\left(y\right)/\left(1+q^{-1}\,{\left(y\right)}^{2}\right)$$
$$\leq \frac{d}{d\,y}\,q^{-1}\,\left(y\right)\leq 1/q^{-1}\,\left(y\right),$$

$$q^{-1}\,{\left(y\right)}^{2}\geq y-q\,\left(1\right)+1,$$
$$q^{-1}\,{\left(y\right)}^{2}$$
≥y-q⁢(1)+1,
for y≥q⁢(1)≈1.841,
for y≥q⁢(1)≈1.841,

$$q^{-1}\,{\left(y\right)}^{2}\leq 2\,\left(y-\log\left(2\right)\right),$$
$$q^{-1}\,{\left(y\right)}^{2}$$
≤2⁢(y-log⁡(2)),
for y≥q⁢(0)=log⁡(2),
for y≥q⁢(0)=log⁡(2),

which we now establish. By implicit differentiation and from the properties of Y noted after Eq. 57, ${\frac{d}{d\,y}\,q^{-1}\,\left(y\right)|}_{y=q\,\left(x\right)}=Y\,\left(x\right)\in \left[x/\left(1+x^{2}\right),1/x\right]$. Therefore $q^{-1}\,\left(y\right)/\left(1+q^{-1}\,{\left(y\right)}^{2}\right)\leq \frac{d}{d\,y}\,q^{-1}\,\left(y\right)\leq 1/q^{-1}\,\left(y\right)$. For y≥log⁡(2), we can integrate $\frac{d}{d\,z}\,q^{-1}\,{\left(z\right)}^{2}=2\,q^{-1}\,\left(z\right)\,\frac{d}{d\,z}\,q^{-1}\,\left(z\right)\leq 2$ from z=log⁡(2) to y to show that $q^{-1}\,{\left(y\right)}^{2}=q^{-1}\,{\left(y\right)}^{2}-q^{-1}\,{\left(\log\left(2\right)\right)}^{2}\leq 2\,\left(y-\log\left(2\right)\right)$, making use of the identity $q^{-1}\,\left(\log\left(2\right)\right)=0$. Consider y,z≥q⁢(1). Since $q^{-1}\,\left(z\right)$ and $0\leq x\mapsto x^{2}/\left(1+x^{2}\right)$ are monotone increasing, $q^{-1}\,{\left(z\right)}^{2}/\left(1+q^{-1}\,{\left(z\right)}^{2}\right)\geq q^{-1}\,{\left(q\,\left(1\right)\right)}^{2}/\left(1+q^{-1}\,{\left(q\,\left(1\right)\right)}^{2}\right)=1/2$, so the integral of $\frac{d}{d\,z}\,q^{-1}\,{\left(z\right)}^{2}$ from z=q⁢(1) to y with the lower bound on $\frac{d}{d\,z}\,q^{-1}\,\left(z\right)$ gives $q^{-1}\,{\left(y\right)}^{2}-q^{-1}\,{\left(q\,\left(1\right)\right)}^{2}=q^{-1}\,{\left(y\right)}^{2}-1\geq y-q\,\left(1\right)$.

From the inequality $\frac{d}{d\,y}\,q^{-1}\,\left(y\right)\leq 1/q^{-1}\,\left(y\right)$ in Eq. 91, integration and monotonicity, for 0≤z≤δ,

$$q^{-1}\,\left(\alpha -z\right)\geq q^{-1}\,\left(\alpha \right)-\frac{z}{q^{-1}\,\left(\alpha -\delta \right)}\leq q^{-1}\,\left(\alpha \right)\,\left(1-\frac{z}{q^{-1}\,{\left(\alpha -\delta \right)}^{2}}\right),$$
$$q^{-1}\,\left(\alpha -z\right)$$
$$\geq q^{-1}\,\left(\alpha \right)-\frac{z}{q^{-1}\,\left(\alpha -\delta \right)}\leq q^{-1}\,\left(\alpha \right)\,\left(1-\frac{z}{q^{-1}\,{\left(\alpha -\delta \right)}^{2}}\right),$$

$$q^{-1}\,\left(\alpha +z\right)\leq q^{-1}\,\left(\alpha \right)+\frac{z}{q^{-1}\,\left(\alpha -\delta \right)}\geq q^{-1}\,\left(\alpha \right)\,\left(1+\frac{z}{q^{-1}\,{\left(\alpha -\delta \right)}^{2}}\right).$$
$$q^{-1}\,\left(\alpha +z\right)$$
$$\leq q^{-1}\,\left(\alpha \right)+\frac{z}{q^{-1}\,\left(\alpha -\delta \right)}\geq q^{-1}\,\left(\alpha \right)\,\left(1+\frac{z}{q^{-1}\,{\left(\alpha -\delta \right)}^{2}}\right).$$

To determine the relative error, write $\delta^{\prime }=\delta /\alpha$ to obtain the interval inclusion

$$q^{-1}\,\left(\alpha \,\left(1+\delta^{\prime }\,\left[-1,1\right]\right)\right)\subseteq q^{-1}\,\left(\alpha \right)\,\left(1+\frac{\alpha \,\delta^{\prime }}{q^{-1}\,{\left(\alpha \,\left(1-\delta^{\prime }\right)\right)}^{2}}\,\left[-1,1\right]\right).$$

For $\alpha \,\left(1-\delta^{\prime }\right)>q\,\left(1\right)$, the interval relationship can be weakened to

$$q^{-1}\,\left(\alpha \,\left(1+\delta^{\prime }\,\left[-1,1\right]\right)\right)\subseteq q^{-1}\,\left(\alpha \right)\,\left(1+\frac{\alpha \,\delta^{\prime }}{\alpha \,\left(1-\delta^{\prime }\right)-q\,\left(1\right)+1}\,\left[-1,1\right]\right).$$

The relative error on the right-hand side is given by the term multiplying the interval, and can be written as $\alpha \,\delta^{\prime }/\left(\alpha -\left(\alpha \,\delta^{\prime }+q\,\left(1\right)-1\right)\right)$. If $\alpha \,\delta^{\prime }+q\,\left(1\right)-1\leq \alpha /2$, then the relative error is bounded by $2\,\delta^{\prime }$ which is twice the relative error of α. Of course, for the interval bounds to converge, we need α=o⁢(n).

**Proof.**

As in the proof of Theorem 12, consider the parametrized bound $\alpha \leq 2\,n\,{\hat{\theta }}^{2}\,{\left(1-\hat{\theta }\right)}^{2}\,{\left(1-a_{1}\right)}^{2}$, where later we set $a_{1}=3/4$ to match the statement of Theorem 14. From the Chernoff-Hoeffding bound, we get $\varphi \geq a_{1}\,\hat{\theta }$ and $\gamma_{\alpha }\leq \sqrt{\alpha /\left(2\,\hat{\theta }\,\left(1-\hat{\theta }\right)\right)}\leq \sqrt{n\,\hat{\theta }\,\left(1-\hat{\theta }\right)}\,\left(1-a_{1}\right)$.

Define $\tilde{\gamma }=\left(\hat{\theta }-\varphi \right)/\sqrt{\varphi \,\left(1-\varphi \right)/n}$. We start from Eq. 64, rewritten as follows:

$$-\log\left(P_{X}\right)\in n\,\mathrm{KL}\,\left(\hat{\theta }|\varphi \right)+\frac{1}{2}\,\log\left(2\,\pi \right)-\log Y\,\left(\tilde{\gamma }\right)-\frac{1}{2}\,\log\left(\frac{\hat{\theta }\,\left(1-\varphi \right)}{\left(1-\hat{\theta }\right)\,\varphi }\right)$$
$$-\log\left(P_{X}\right)$$
$$\in n\,\mathrm{KL}\,\left(\hat{\theta }|\varphi \right)+\frac{1}{2}\,\log\left(2\,\pi \right)-\log Y\,\left(\tilde{\gamma }\right)-\frac{1}{2}\,\log\left(\frac{\hat{\theta }\,\left(1-\varphi \right)}{\left(1-\hat{\theta }\right)\,\varphi }\right)$$

$$+\left[-\frac{{\mathrm{lE}}_{n}\,\left(\hat{\theta }|\varphi \right)}{n\,\left(\hat{\theta }-\varphi \right)},\frac{1}{12\,n\,\hat{\theta }\,\left(1-\hat{\theta }\right)}\right].$$
$$+\left[-\frac{{\mathrm{lE}}_{n}\,\left(\hat{\theta }|\varphi \right)}{n\,\left(\hat{\theta }-\varphi \right)},\frac{1}{12\,n\,\hat{\theta }\,\left(1-\hat{\theta }\right)}\right].$$

If $\tilde{\gamma }\geq \sqrt{8/\pi }\approx 1.6$, ${\mathrm{lE}}_{n}\,\left(\hat{\theta }|\varphi \right)=1$. For better bounds at small values of $\tilde{\gamma }$, we use the other alternative in the definition of ${\mathrm{lE}}_{n}$, according to which the lower bound in the last interval of Eq. 95 is

$$-\frac{{\mathrm{lE}}_{n}\,\left(\hat{\theta }|\varphi \right)}{n\,\left(\hat{\theta }-\varphi \right)}\geq -\frac{\sqrt{\pi /8}}{\sqrt{n\,\varphi \,\left(1-\varphi \right)}}\geq -\frac{\sqrt{\pi /8}}{\sqrt{n\,a_{1}\,\hat{\theta }\,\left(1-\varphi \right)}}\geq -\frac{\sqrt{\pi /8}}{\sqrt{n\,a_{1}\,\hat{\theta }\,\left(1-\hat{\theta }\right)}}.$$

Next we approximate $n\,\mathrm{KL}\,\left(\hat{\theta }|\varphi \right)$ in terms of $\tilde{\gamma }$ instead of γ. We still write the interval bounds in terms of γ. Let f⁢(x)=KL⁢(φ+x|φ). We are concerned with the range $0\leq x\leq \hat{\theta }-\varphi$, with $\varphi \geq a_{1}\,\hat{\theta }$. We have

$$f^{\left(1\right)}\,\left(x\right)=\log\left(\left(\varphi +x\right)/\varphi \right)-\log\left(\left(1-\varphi -x\right)/\left(1-\varphi \right)\right)$$
$$f^{\left(1\right)}\,\left(x\right)$$
=log⁡((φ+x)/φ)-log⁡((1-φ-x)/(1-φ))

$$f^{\left(2\right)}\,\left(x\right)=\frac{1}{\varphi +x}+\frac{1}{1-\varphi -x}$$
$$f^{\left(2\right)}\,\left(x\right)$$
$$=\frac{1}{\varphi +x}+\frac{1}{1-\varphi -x}$$

$$=\frac{1}{\left(\varphi +x\right)\,\left(1-\varphi -x\right)}$$
$$=\frac{1}{\left(\varphi +x\right)\,\left(1-\varphi -x\right)}$$

$$f^{\left(3\right)}\,\left(x\right)=-\frac{1}{{\left(\varphi +x\right)}^{2}}+\frac{1}{{\left(1-\varphi -x\right)}^{2}}$$
$$f^{\left(3\right)}\,\left(x\right)$$
$$=-\frac{1}{{\left(\varphi +x\right)}^{2}}+\frac{1}{{\left(1-\varphi -x\right)}^{2}}$$

$$=-\frac{1-2\,\left(\varphi +x\right)}{{\left(\varphi +x\right)}^{2}\,{\left(1-\varphi -x\right)}^{2}}$$
$$=-\frac{1-2\,\left(\varphi +x\right)}{{\left(\varphi +x\right)}^{2}\,{\left(1-\varphi -x\right)}^{2}}$$

$$\left|f^{\left(3\right)}\,\left(x\right)\right|\leq \frac{1}{a_{1}^{2}\,{\hat{\theta }}^{2}\,{\left(1-\hat{\theta }\right)}^{2}},$$
$$\left|f^{\left(3\right)}\,\left(x\right)\right|$$
$$\leq \frac{1}{a_{1}^{2}\,{\hat{\theta }}^{2}\,{\left(1-\hat{\theta }\right)}^{2}},$$

yielding

$$\mathrm{KL}\,\left(\varphi +x|\varphi \right)\in \frac{x^{2}}{2\,\varphi \,\left(1-\varphi \right)}+\frac{x^{3}}{6\,a_{1}^{2}\,{\hat{\theta }}^{2}\,{\left(1-\hat{\theta }\right)}^{2}}\,\left[-1,1\right],$$

and with $x=\tilde{\gamma }\,\sqrt{\varphi \,\left(1-\varphi \right)/n}=\gamma \,\sqrt{\hat{\theta }\,\left(1-\hat{\theta }\right)/n}$,

$$n\,\mathrm{KL}\,\left(\hat{\theta }|\varphi \right)\in \frac{{\tilde{\gamma }}^{2}}{2}+\frac{\gamma^{3}}{6\,a_{1}^{2}\,\sqrt{n\,\hat{\theta }\,\left(1-\hat{\theta }\right)}}\,\left[-1,1\right].$$

For the fourth term on the right-hand side of Eq. 95,

$$\frac{d}{d\,x}\,\log\left(\frac{\hat{\theta }\,\left(1-\hat{\theta }+x\right)}{\left(1-\hat{\theta }\right)\,\left(\hat{\theta }-x\right)}\right)=\frac{1}{1-\hat{\theta }+x}+\frac{1}{\hat{\theta }-x}=\frac{1}{\left(1-\hat{\theta }+x\right)\,\left(\hat{\theta }-x\right)},$$

whose absolute value is bounded by $1/\left(a_{1}\,\hat{\theta }\,\left(1-\hat{\theta }\right)\right)$ for x in the given range. Thus

$$\log\left(\frac{\hat{\theta }\,\left(1-\varphi \right)}{\left(1-\hat{\theta }\right)\,\varphi }\right)\in \frac{\gamma }{a_{1}\,\sqrt{n\,\hat{\theta }\,\left(1-\hat{\theta }\right)}}\,\left[-1,1\right].$$

Since $P_{X}\leq P_{\mathrm{CH}}$, we can also use the bound $\gamma \leq 2\,\sqrt{3/5}\,\sqrt{\alpha }$ obtained in the proof of Theorem 12. Substituting $a_{1}=3/4$ as needed, the equation to solve is now

$$\alpha \in \frac{{\tilde{\gamma }}^{2}}{2}+\frac{1}{2}\,\log\left(2\,\pi \right)-\log Y\,\left(\tilde{\gamma }\right)$$
α
$$\in \frac{{\tilde{\gamma }}^{2}}{2}+\frac{1}{2}\,\log\left(2\,\pi \right)-\log Y\,\left(\tilde{\gamma }\right)$$

$$+\frac{8}{\sqrt{15}}\,\frac{\sqrt{\alpha }}{\sqrt{n\,\hat{\theta }\,\left(1-\hat{\theta }\right)}}\,\left[-1,1\right]+\frac{64}{15\,\sqrt{15}}\,\frac{{\sqrt{\alpha }}^{3}}{\sqrt{n\,\hat{\theta }\,\left(1-\hat{\theta }\right)}}\,\left[-1,1\right]$$
$$+\frac{8}{\sqrt{15}}\,\frac{\sqrt{\alpha }}{\sqrt{n\,\hat{\theta }\,\left(1-\hat{\theta }\right)}}\,\left[-1,1\right]+\frac{64}{15\,\sqrt{15}}\,\frac{{\sqrt{\alpha }}^{3}}{\sqrt{n\,\hat{\theta }\,\left(1-\hat{\theta }\right)}}\,\left[-1,1\right]$$

$$+\left[-\frac{\sqrt{\pi /6}}{\sqrt{n\,\hat{\theta }\,\left(1-\hat{\theta }\right)}},\frac{1}{12\,n\,\hat{\theta }\,\left(1-\hat{\theta }\right)}\right].$$
$$+\left[-\frac{\sqrt{\pi /6}}{\sqrt{n\,\hat{\theta }\,\left(1-\hat{\theta }\right)}},\frac{1}{12\,n\,\hat{\theta }\,\left(1-\hat{\theta }\right)}\right].$$

The sum of the first three terms evaluates to $q\,\left(\tilde{\gamma }\right)$. The remaining terms are now independent of γ and are of order $1/\sqrt{n}$. They can be merged by means of common bounds using $2\,n\,\hat{\theta }\,\left(1-\hat{\theta }\right)\geq \sqrt{n\,\hat{\theta }\,\left(1-\hat{\theta }\right)}$, since $n\,\hat{\theta }\,\left(1-\hat{\theta }\right)\geq 1/2$ for our standing assumptions that n≥1 and $\hat{\theta }\,n$ is an integer different from 0 and n. Consequently, $12\,n\,\hat{\theta }\,\left(1-\hat{\theta }\right)\geq 6\,\sqrt{n\,\hat{\theta }\,\left(1-\hat{\theta }\right)}\geq \sqrt{6/\pi }\,\sqrt{n\,\hat{\theta }\,\left(1-\hat{\theta }\right)}$. The interval bounds then combine conservatively to

$$\frac{\sqrt{\pi /6}+8\,\sqrt{\alpha }/\sqrt{15}+64\,{\sqrt{\alpha }}^{3}/\left(15\,\sqrt{15}\right)}{\sqrt{n\,\hat{\theta }\,\left(1-\hat{\theta }\right)}}.$$

We can now write

$$\alpha \in q\,\left(\tilde{\gamma }\right)+\frac{\sqrt{\pi /6}+8\,\sqrt{\alpha }/\sqrt{15}+64\,{\sqrt{\alpha }}^{3}/\left(15\,\sqrt{15}\right)}{\sqrt{n\,\hat{\theta }\,\left(1-\hat{\theta }\right)}}\,\left[-1,1\right],$$

which holds iff

$$q\,\left(\tilde{\gamma }\right)\in \alpha \,\left(1+\frac{64\,\sqrt{\alpha }/\left(15\,\sqrt{15}\right)}{\sqrt{n\,\hat{\theta }\,\left(1-\hat{\theta }\right)}}\,\left[-1,1\right]\right)+\frac{\sqrt{\pi /6}+8\,\sqrt{\alpha }/\sqrt{15}}{\sqrt{n\,\hat{\theta }\,\left(1-\hat{\theta }\right)}}\,\left[-1,1\right].$$

By monotonicity of q and extending $q^{-1}$ to negative arguments as mentioned in the statement of Theorem 14 if necessary, the constraint is equivalent to

$$\tilde{\gamma }\in q^{-1}\,\left(\alpha \,\left(1+\frac{64\,\sqrt{\alpha }/\left(15\,\sqrt{15}\right)}{\sqrt{n\,\hat{\theta }\,\left(1-\hat{\theta }\right)}}\,\left[-1,1\right]\right)+\frac{\sqrt{\pi /6}+8\,\sqrt{\alpha }/\sqrt{15}}{\sqrt{n\,\hat{\theta }\,\left(1-\hat{\theta }\right)}}\,\left[-1,1\right]\right).$$

For α>log⁡(2), we know that $\tilde{\gamma }>0$, so we can add max⁡(0,…) as in the theorem statement.

To determine the interval equation for γ, we have $\gamma =\tilde{\gamma }\,\sqrt{\varphi \,\left(1-\varphi \right)/\left(\hat{\theta }\,\left(1-\hat{\theta }\right)\right)}$. We use the first-order remainder to bound the factor on the right-hand side. For this consider the numerator, and write $g\,\left(x\right)=\sqrt{\left(\hat{\theta }-x\right)\,\left(1-\hat{\theta }+x\right)}$ with $0\leq x\leq \hat{\theta }-\varphi$. We have

$$g^{\left(1\right)}\,\left(x\right)=\frac{2\,\left(\hat{\theta }-x\right)-1}{2\,\sqrt{\left(\hat{\theta }-x\right)\,\left(1-\hat{\theta }+x\right)}},$$
$$g^{\left(1\right)}\,\left(x\right)$$
$$=\frac{2\,\left(\hat{\theta }-x\right)-1}{2\,\sqrt{\left(\hat{\theta }-x\right)\,\left(1-\hat{\theta }+x\right)}},$$

$$\left|g^{\left(1\right)}\,\left(x\right)\right|\leq \frac{1}{2\,\sqrt{a_{1}\,\hat{\theta }\,\left(1-\hat{\theta }\right)}}$$
$$\left|g^{\left(1\right)}\,\left(x\right)\right|$$
$$\leq \frac{1}{2\,\sqrt{a_{1}\,\hat{\theta }\,\left(1-\hat{\theta }\right)}}$$

$$=\frac{1}{\sqrt{3\,\hat{\theta }\,\left(1-\hat{\theta }\right)}},$$
$$=\frac{1}{\sqrt{3\,\hat{\theta }\,\left(1-\hat{\theta }\right)}},$$

$$g\,\left(x\right)\in \sqrt{\hat{\theta }\,\left(1-\hat{\theta }\right)}+\frac{x}{\sqrt{3\,\hat{\theta }\,\left(1-\hat{\theta }\right)}}\,\left[-1,1\right].$$
g⁢(x)
$$\in \sqrt{\hat{\theta }\,\left(1-\hat{\theta }\right)}+\frac{x}{\sqrt{3\,\hat{\theta }\,\left(1-\hat{\theta }\right)}}\,\left[-1,1\right].$$

With $x=\gamma \,\sqrt{\hat{\theta }\,\left(1-\hat{\theta }\right)/n}$ and the bound of $\gamma \leq 2\,\sqrt{3/5}\,\sqrt{\alpha }$, we get

$$\gamma \in \tilde{\gamma }\,\left(1+\frac{2\,\sqrt{\alpha }/\sqrt{5}}{\sqrt{n\,\hat{\theta }\,\left(1-\hat{\theta }\right)}}\,\left[-1,1\right]\right).$$

The theorem follows by composing this constraint with Eq. 106. ∎
